# Automated thermal desorption-gas chromatography/mass spectrometry for screening of hazardous chemicals in cotton and cotton blend garments—analytical challenges

**DOI:** 10.1007/s00216-025-05993-y

**Published:** 2025-07-08

**Authors:** Tim Åström, Conny Östman, Ioannis Sadiktsis, Maria-Ximena Ruiz-Caldas, Ulrika Nilsson

**Affiliations:** Department of Chemistry, Stockholm University, 11418 Stockholm, Sweden

**Keywords:** ATD-GC/MS, Cotton, Cellulose, Textile chemicals, Quantitative analysis, Clothing

## Abstract

The global production of textiles involves large amounts of health-hazardous chemicals, constituting possible health risks since residues usually remain in the finished garments. In the present study, a recently published ATD-GC/MS methodology for screening synthetic textiles is further extended to cotton and cotton blend materials. Different textile materials with a high content of cotton were found to exhibit large variations in adsorption strength for a number of chemicals frequently detected in textiles. This was shown to strongly influence the thermal desorption efficiency in ATD-GC/MS. By using absolute response factors from appropriate internal standards spiked directly onto the textile samples, the effects from these differences could be minimized. In this way, accurate quantification was made possible regardless of textile composition, and quantification of native textile chemicals in garments made with the ATD-GC/MS method agreed well with an offline method based on solvent extraction and GC/MS analysis. The ATD-GC/MS method has now been shown to be applicable for quantitative screening of at least 75% of all the clothing textiles on the retail market. The simplified quantification method makes it suitable for screening large numbers of samples. For all fiber materials investigated, the method limit of detection, using only 20 mg of textile, is at least 100 times lower than the current EU regulation for quinoline and a number of toxic arylamines.

**Graphical abstract:**

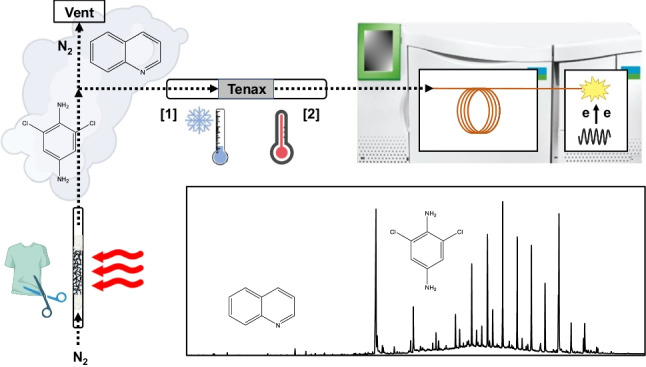

**Supplementary Information:**

The online version contains supplementary material available at 10.1007/s00216-025-05993-y.

## Introduction

The global textile industry faces significant environmental challenges [1]. Emerging consumer trends give rise to increased textile consumption, as well as shorter lifespan of garments, creating a systematic overproduction of textile apparel [2]. Global annual fiber production reached 116 million metric tonnes in 2022 and is expected to rise to 147 million metric tonnes by 2030 [3]. Approximately 60% of the global fiber production is destined for the fashion industry [3], and consisted in 2022 of 65% synthetic, 27% natural, 6% artificial cellulosic, and 2% other fibers. A common practice in the textile industry is fiber blending, which enhances the mechanical properties of fabrics and, in some cases, reduces costs [4].

Among synthetic fabrics, polyester was the predominant material with 54% of the global production, followed by polyamide with 5%. Among natural fibers, cotton was the most common with 22% of the global production, while wool only accounted for 1% of the market. Artificial cellulosic fabrics include materials such as viscose, acetate, lyocell, modal, and cupro. Fabrics made of recycled materials constituted in total 7.9% of the global fiber production in 2022. However, less than 1% of this recycled material originated from pre- and/or post-consumer garments, with 99% recycled from polyethylene terephthalate plastic bottles. Recycled cotton is estimated to be 300,000 metric tonnes, accounting for only 1% of the annual cotton production and around 1.4% of organic cotton. This figure is expected to increase significantly in the coming years [5].

For a sustainable textile production, it is important to not only consider the fiber material per se but also the content of possible health-hazardous and environmentally hazardous chemicals. Cotton, often perceived as a safe and ecological natural material, consists of 88–96.5% α-cellulose and around 1% of proteins, organic acids, inorganic substances, and other types of sugars [6]. The remaining group of natural substances found in cotton fibers includes certain types of waxes, fats, and resins, commonly named cotton waxes. Typical compounds in this class are fatty acids, sterols, glycerols, glycols, long-chain aliphatic alcohols, α- and β-amyrins, esters of fatty acids, etc. [6–8]. Most of these non-cellulosic compounds are removed during fabric production, increasing the cellulose content to over 99% [9]. However, many chemicals may also be added during fabric production, which may remain in the finished product both intentionally and unintentionally. For instance, functional chemicals may be used to reduce the friction between the fibers, yarns, or fabrics, and lubricants are commonly added during weaving, sewing, and knitting processes [10]. Other examples of purposely added chemicals include dyes, pigments, and water-repelling fluorinated chemicals.

Several hundred unintentionally added chemicals, possibly or proven hazardous, have been identified in various types of cloth and textile materials. Examples are heavy metals [11–13], free arylamines as residues from azo dye syntheses [14–21], quinoline, isoquinoline, substituted methyl and dimethyl quinolines [20, 22–25], benzothiazoles and benzotriazoles [25–27], dioxins and furans [28], polychlorinated biphenyls (PCBs) [29, 30], organophosphorus pesticides (OPPs) [31], halogenated flame retardants [32–34], phthalates [35], glycol solvents [36], formaldehyde [37–40] C_10_-C_16_ alkanes, C_3_ alkylbenzenes, and C_2_-C_10_ aldehydes [41].

A solvent-free analytical method has previously been published, based on automated thermal desorption (ATD) coupled to GC/MS, for screening of health-hazardous chemicals in textile garments [19]. The method is intended for implementation within the production chain in order to detect and prevent hazardous chemicals from reaching the retail market. It has been evaluated for screening of synthetic fabrics, mainly polyester and polyester blends, detecting and quantifying, e.g., quinolines, arylamines and halogenated nitrobenzenes. The ATD-GC/MS method was found to yield reproducible results with detection limits sufficiently low to meet or, in most cases, be well below current EU regulations [42, 43]. Examples of textile chemicals included in REACH are azo colorants, which can release any of 22 restricted arylamines at levels > 30 µg/g. Quinoline is another compound, restricted to 50 µg/g in textiles. A number of phthalates are prohibited if their total concentration exceeds 1000 µg/g. Compared with offline GC/MS that requires prior solvent extraction, the ATD-GC/MS method is solvent-free, less laborious, and has a higher sample throughput, requiring only around 40 min per sample. Another strength of the ATD-GC/MS is its ability to measure those semi-volatile organic textile chemicals most prone to migrate from the garments and be absorbed by human skin, i.e., compounds not covalently bound to the fibers, having molecular weights below 1000 Da and log*P* around 2–4.

The present study aimed to further develop and evaluate ATD-GC/MS for screening of chemicals present in textile materials made of cotton as well as cotton blends. Unlike polyester and other synthetic fibers, cotton fibers naturally have a complex hierarchical structure, consisting of several parts, from the central hollow canal, via the lumen wall, the secondary walls, the winding layer, and the primary wall, out to the outer waxy cuticle layer of proteins and pectins [44]. The different layers of the fiber structure contain fibrils of various sizes, and between them are variously sized pores/capillary spaces. This, coupled with the thread density and entwinement of the fabric, could affect its interfacial properties. Altogether, this may affect the performance of the ATD-GC/MS for cotton analysis. To the best of our knowledge, this is the first study of ATD-GC/MS for qualitative and quantitative analysis of cotton materials. The present study includes an investigation of cotton in terms of porosity, thread density, and adsorption properties.

## Materials and methods

### Chemicals and materials

Methanol, acetone (both of HPLC grade), toluene, and dichloromethane (puriss) were obtained from VWR (Radnor, PA, USA). Deactivated (silanized) glass wool of pesticide grade was obtained from Supelco (Bellefonte, PA). Internal standards (IS) and reference compounds used for evaluation of the ATD-GC/MS method are listed in the Electronic Supplementary Material, Table S1. SPE cartridges containing 500 mg of porous graphitized carbon (Carbograph 5, mesh size 120/400, surface area 240 m^2^/g) were purchased from LARA Srl (Formello, Italy). The cold trap in the ATD was packed with Tenax-TA^®^, which was purchased from Perkin Elmer Inc (Waltham, MA, USA).

### Standard solutions

Individual stock solutions of the reference compounds, each at approx. 1 mg/mL, were prepared in acetonitrile, purged with argon, and stored at − 24 °C. Working external standard solutions including all analytes and IS, each at approx. 16 ng/µL in acetonitrile, were prepared fresh every week from the stock solutions and stored at − 24 °C. No degradation of the reference compounds was observed during this period of time, except for 2,6-dichloro-1,4-phenylenediamine and 3,5-dibromo-1,2-phenylenediamine, which started to show some minor degradation after one week. This was not further studied. The IS solution used for spiking of samples contained 16 ng/µL each of quinoline-d_7_, 2-methylbenzothiazole, 3-nitroaniline-d_4_, benzophenone-d_10_, 4-nitroaniline-^15^N_2_, 1-bromo-2,4-dinitrobenzene-d_3_, diethyl phthalate-d_4_, and bis(2-ethylhexyl) phthalate-d_4_. Quinoline-d_7_ was used for quantification of quinolines and benzothiazole, 3-nitroaniline-d_4_ and 4-nitroaniline-^15^N_2_ for arylamines, and 1-bromo-2,4-dinitrobenzene-d_3_ for halogenated dinitrobenzenes. A solution of *n*-eicosane-d_42_, 40.0 ng/µL, was also prepared as a volumetric IS solution for the offline GC/MS analyses.

### Textile samples

#### Textile materials spiked with reference compounds

The ATD/GC–MS performance for cotton analysis was initially investigated using 100% cotton materials spiked with the external standard compounds and compared with spiked compounds on 100% polyester. The cotton samples were: Cotton-1, a pair of gloves made of 100% cotton (VWR Radnor, PA, catalogue no.: 113–7356) and Cotton-2, a cleaning cloth for instruments (Agilent technologies, Palo Alto, CA, USA). Optical and electron microscope images of these materials are found in Fig. 5. The polyester material was from a white bathrobe, pre-conditioned in the ATD at 200 °C for 20 min. Both polyester and cotton were further compared for use as “External Standard Textile Matrix” for analyses of eight spiked garments of different cotton and cotton blend compositions (Table 1). These eight garments were obtained from the Swedish retail market and were chosen due to low contents of chemical residues. Optical and electron microscope images of these samples are found in Figs. SI–13. Blank correction was performed using corresponding non-spiked textiles.

**Table 1:** Textile samples spiked with external standard and used for evaluation of the ATD system

Sample ID	Color	Composition	Country of origin	Type of garment
S1	White	100% cotton	Bangladesh	T-shirt
S2	White	100% cotton	Bangladesh	T-shirt
S3	White	100% cotton	India	T-shirt
S4	White	100% cotton	Pakistan	Glove
S5	White	95% cotton, 5% elastane	India	T-shirt
S6	White	95% cotton, 5% elastane	Bangladesh	Bodywear
S7	White	75% cotton, 23% polyamide, 2% elastane	Turkey	Socks
S8	White	38% cotton, 40% polyester, 16% polyamide, 6% acrylics	Myanmar	Blouse

#### Clothing samples for analysis with both ATD-GC/MS analysis and offline GC/MS

Four cotton-based infant clothing garments (non-spiked), all obtained from the Swedish retail market, were analyzed for the content of native compounds with both ATD-GC/MS and solvent extraction followed by offline GC/MS (SE-GC/MS) (see Table 2). Each garment was analyzed in triplicate, and quantification was performed using one-point calibration within the tested linear range.

**Table 2:** Textile samples analyzed with both ATD-GC/MS and SE-GC/MS

Sample ID	Color	Composition	Country of origin	Type of garment
Textile-1	Red	95% cotton, 5% elastane	Bangladesh	Body dress
Textile-2	Red	100% cotton	Bangladesh	Blouse
Textile-3	Blue	100% cotton	Bangladesh	Baby pajamas
Textile-4	Black	100% cotton	India	Baby pajamas

### ATD-GC/MS analysis

Around 2 g of each textile sample was prepared by cutting it into 1 × 1 mm pieces using a pair of scissors. The pieces were mixed, and around 20 mg of this material was placed in a 3-mm desorption glass tube (Perkin Elmer), between two pre-conditioned (300 °C for 10 min) silanized glass wool (Supelco) plugs. A volume of 10 µL IS solution, including *n*-eicosane-d_42_, was spiked on the sample inside the tube (each compound approx. 160 ng) and left to dry for 3 h prior to desorption.

#### The ATD-GC/MS instrument

**Fig. 1:**
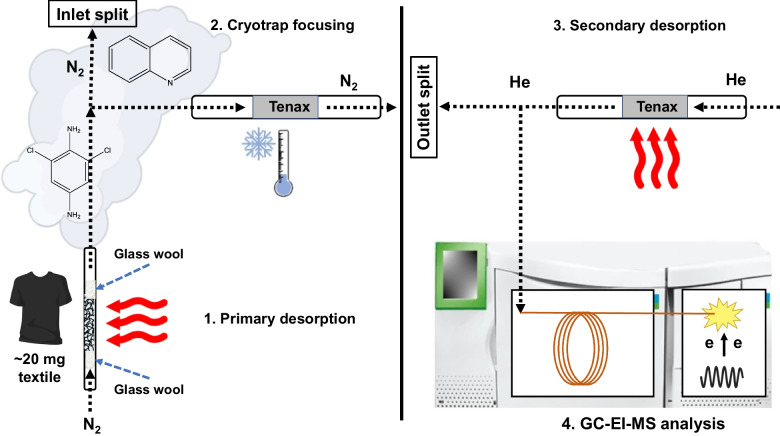
Schematic illustration of the ATD-GC/MS methodology

The instrument consisted of a TurboMatrix 350 automated thermal desorber (ATD) coupled online to a Clarus® 680 gas chromatograph/Clarus® SQ 8 T mass spectrometer (Perkin Elmer). The ATD programme was based on our previously developed method [19], using desorption at 175 °C for 10 min, Tenax-TA® as trap adsorbent and a cold trap temperature at − 10 °C. Some minor modifications to the method were made; trap desorption temperature was set to 260 °C, and both transfer line and transfer valve temperatures were set to 270 °C. An inlet and an outlet split were utilized to prevent sample overload and instrument contamination, resulting in a theoretical total maximum transfer of approximately 6% of the desorbed amount of analyte to the GC. The inlet split (49 mL/min) was used during the sample desorption (desorption flow: 50 mL/min), resulting in a 1/49 dilution ratio on the cold trap. The higher inlet split allows for higher desorption flow rates without compromising the trapping efficiency of the cold trap. During the sample transfer from the cold trap to the analytical column, an outlet split of 10 mL/min was used to reduce the background and potential bleeding from the stationary phase of the cold trap. The system is illustrated in Fig. 1.

The helium carrier gas with a purity of 99.999% (Strandmöllen AB, Ljungby, Sweden) was further purified with an in-line gas filter (RMSH-2, Agilent Technologies, Palo Alto, CA, USA). The carrier gas flow was 1.3 mL/min, resulting in a total trap desorption flow of 11.3 mL/min. The GC column was a DB-5MS, 40 m × 0.25 mm i.d., film thickness = 0.25 µm (Agilent Technologies, Palo Alto, CA, USA) with an integrated 10-m guard column. The GC oven program started at 90 °C, kept for 0.5 min, followed by a temperature ramp of 10 °C/min up to 325 °C held for 12 min, resulting in a total run time of 40 min. Except for the first sample in a series, the total sample run time was 40 min, with 10 min of desorption overlapping with the next GC program, i.e., the run time for the first sample was 50 min. The MS transfer line temperature was set to 250 °C and the MS ion source to 230 °C, and electron ionization (EI) was applied using an electron energy of 70 eV, with the filament turned on after 5.0 min of run time. For identification of compounds, the MS was operated in full scan mode between *m/z* 50 and 500 with 200 ms scan time with an interscan delay of 50 ms. For quantification, the ion trace of the most intense ion was used (Q1), while intensity ratios of at least two fragment ions were used for identification (see Table S2 in Electronic Supplementary Material).

### Preparation of solvent extracts for offline GC/MS analysis

Each textile was prepared by cutting it into small pieces of approx. 3 × 3 mm. Triplicate samples of approximately 20 mg were prepared and spiked with around 20 µg each of individual IS components. The samples were then left to dry for 3 h prior to extraction. The solvent extraction was performed during 10 min using ultrasonication in 10 mL of dichloromethane at 35 °C (USC 600 TH, VWR, Radnor, PA, USA). The extraction procedure was repeated twice with fresh solvent. The extracts were pooled, and the volume was reduced to approx. 1 mL at 40 °C using a gentle stream of N_2_. Non-volatile co-extracted disperse dyes were removed with SPE using Carbograph 5 [20], which was conditioned with 10 mL of methanol followed by 5 mL of dichloromethane/toluene (8:2 v/v). The sample extracts were loaded onto the cartridge and eluted by gravity flow using 15 mL of dichloromethane/toluene (8:2). The eluate was concentrated to approx. 1 mL at 40 °C using a gentle stream of N_2_. Prior to GC/MS analysis, 2.00 µg of volumetric IS (*n*-eicosane-d_42_) was added to each vial.

All fifty-six compounds were used in the offline GC/MS analyses of the solvent extracts, except bis(2-ethylhexyl) phthalate, detected in all extracts as a contaminant originating from the laboratory environment. The absolute recoveries from cotton were found to be lower than 30% for most of the investigated compounds, although most compounds had a corrected recovery ranging between 85 and 115% (Table S3).

### Offline GC/MS

The GC/MS system for the offline analyses consisted of a 6890 N gas chromatograph, a 7683 autosampler, and a 5975 C mass spectrometer (Agilent Technologies, Palo Alto, CA, USA). The GC was equipped with a programmable temperature vaporizer (PTV) injector (Gerstel GmbH & Co, Mulheim an der Ruhr, Germany), programmed as follows: 90 °C for 0.5 min followed by a ramp of 700 °C/min up to 300 °C, which was held for 8 min. The GC oven program was as follows: 90 °C for 0.5 min, followed by a ramp of 10 °C/min up to 325 °C, which was kept for 15 min, resulting in a total run time of 43 min [20]. An Elite-35 ms column (Perkin Elmer, Waltman, MA, USA, 35% diphenyl, 30 m, 0.25 mm i.d., 0.25 µm film thickness) was used. This column is slightly more polar than the DB-5 stationary phase used for ATD-GC/MS, but was chosen since it had been used in the previously described method [16]. The MS was operated in EI mode with an electron energy of 70 eV. The MS source temperature was set to 250 °C, the transfer line to 325 °C, and the quadrupole analyzer to 200 °C. The acquisition was performed in selected ion monitoring mode. A detailed summary of the qualifier and quantifier ions is presented in Table S2.

### Evaluation of ATD-GC/MS performance

Thermal desorption efficiencies were evaluated, as well as linearities of responses, method detection/quantification limits (MDL/MQL), repeatability, carry-over, and method accuracy. The instrumental LOD/LOQ were estimated using responses from triplicate injections of diluted standard solutions. MDL/MQL values were calculated from the instrumental LOD/LOQ based on 20 mg samples. The repeatability over time was investigated by analyzing spiked materials after 6, 9, 12, 15, 18, 21, 24, and 27 h, as shown in Table S4. No carry-over effects from the cold trap or other parts of the instrument were observed.

### Wettability of cotton

The sorption properties of Cotton-1 and Cotton-2 were investigated using the Washburn method, which allows for determining the contact angle, *θ*, of porous materials based on measured sorption velocities [45]. The Washburn equation describes the rise of a liquid inside a tube due to capillary forces. The modified Washburn equation, used for porous materials, is given by:

$$\text{cos}\,\theta =\frac{{m}^{2}}{t}\cdot \frac{\eta }{{\rho }^{2}\gamma C}$$

where ${m}^{2}/t$ represents the sorption velocity, C is the capillary constant, and $\eta$, $\rho$, and $\gamma$ correspond to the liquid viscosity, density, and surface tension, respectively.

The sorption velocities (g^2^/s) of water, ethanol, hexane, and acetonitrile were measured using a force tensiometer (K100, KRÜSS, Germany) with a Washburn sample holder (SH0820, KRÜSS, Germany) and a 13 mm filter disk. Approximately 1 g of cotton fabric, cut into one or two stripes of 3 cm in width, was placed inside the sample holder. The holder was then hung in the tensiometer, and its lower part was brought into contact with the liquid surface. Upon contact with the liquid, the measurement was triggered, and the instrument started recording the mass of the sorbed liquid as a function of time. The sorption velocity was calculated automatically by the software from the linear region of the absorption curve. The test was repeated in triplicate for each cotton-liquid combination. The temperature during the measurement ranged from 23 to 24 °C.

The capillary constant of each cotton fabric (C), a dimensionless factor that characterizes the interaction between a liquid and a cotton fabric, was calculated using the sorption rate of hexane, assuming complete wettability (*θ* = 0.0°) due to the low surface tension of hexane. The physical properties of the liquids used are listed in Table 3.

**Table 3:** Physical properties of liquids at 25 °C used for contact angle measurements

Liquid	Viscosity (mPa s)	Density (g/cm^3^)	Surface tension (mN/m)			Ratio polar to dispersive component
			Total	Polar component	Dispersive component	
Water	0.890	0.997	72.8	51.0	21.8	2.3
Ethanol	1.10	0.7858	22.3	4.6	17.7	0.3
Hexane	0.313	0.659	18.4	18.4	0	0.0
Acetonitrile	0.344	0.786	29.3	19.3	8.8	2.2

Data obtained from the Krüss test liquid database and [46]

### Aspect of fabrics

Optical microscopy images were captured using a USB optical microscope (Dino-Lite AM73915MZT, Dino-Lite Digital Microscope) at × 25 magnification. Scanning Electron Microscopy (SEM) was performed using a HITACHI TM-3000 (Germany) SEM with a 5-kV electron beam at × 80 or × 100 magnification.

The fabric bulk density (${\uprho }_{\text{b}}$) of Cotton-1 and Cotton-2 was estimated by dividing the weight of fabric samples by their volume. The volume was calculated using the area of the sample, measured with ImageJ, and its thickness, measured using a thickness gauge. The thickness was measured ten times, and five samples were weighed and photographed for mass and area measurements. The fabric porosity was determined using the following equation:

$$\text{Porosity }\left[{\%}\right]=1-\frac{{\uprho }_{\text{b}}}{{\uprho }_{\text{s}}}$$

where ${\uprho }_{\text{s}}=1617\text{ kg}/{\text{m}}^{3}$ is the true density of cotton fibers [47].

## Results and discussion

The ATD-GC/MS method has previously been shown to release the investigated textile chemicals in polyester close to 100% at a desorption temperature of 175 °C. A good performance in terms of detection limits and reproducibility was obtained for both pure polyester and polyester mixed with other synthetic fibers [19]. Cotton and cotton blends, on the other hand, are different regarding physical and chemical properties, as mentioned in the Introduction. This would most likely have an influence on the thermal desorption efficiency and could affect the performance of the method.

Throughout the present study, ATD-GC/MS analysis of polyester was used as a benchmark, due to 100% desorption of spiked analyte amounts and thus defining a 100% response for all compounds at a desorption temperature of 175 °C, as shown in Table S5. All analyses included *n*-eicosane-d_42_ since this compound was shown to be fully desorbed and exhibit the same response irrespective of textile composition, and the relative responses (analyte/*n*-eicosane-d_42_) were thus used to compare the different materials.

All MS analyses were performed in full scan mode since one of the aims of the ATD-GC/MS method is to enable both non-target screening and quantitative target analysis within the same run.

### Examination of parameters affecting the ATD-GC/MS analysis

#### The cotton material

**Fig. 2:**
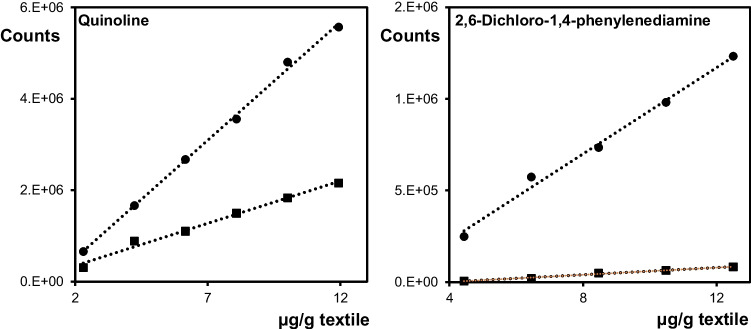
Detector response in the ATD-GC/MS for quinoline and 2,6-dichloro-1,4-phenylenediamine spiked with different amounts added to 20 mg of polyester and cotton (Cotton-1), respectively. ●  = polyester, ■ = cotton

When performing solvent extraction of the investigated compounds spiked on Cotton-1, a material made of 100% cotton, a recovery level below 30% was obtained for most of the compounds, although with a high repeatability. The low recoveries are in strong contrast to the polyester material, which gave a yield close to 100% for almost all of these compounds. A similar behavior was also observed when using the ATD-GC/MS. A majority of the spiked analytes, especially the nitrogen-containing, exhibited a considerably lower ATD-GC/MS response when compared to the polyester material. In Fig. 2, this is exemplified by quinoline and 2,6-dichloro-1,4-phenylenediamine. The yield is high, around 100% when spiked to polyester, the upper curve, and lowest for Cotton-1.

**Fig. 3:**
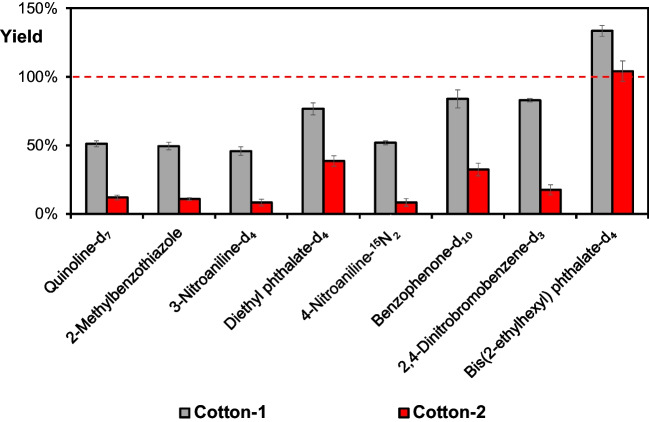
Desorption yields (%) from two cotton matrices for the internal standards representing the different investigated chemical classes of compounds. The compounds are presented in GC retention order. Gray = Cotton-1, red = Cotton-2 (*n* = 3)

In Fig. 3, the yields are shown for the used internal standards, displayed to represent the different investigated analyte classes on both Cotton-1 and another material of 100% cotton fibers, Cotton-2. As shown in the figure, the desorption behavior of the compounds shows large differences between the materials.

There are also large differences between the various compounds when desorbed from the same cotton material, exemplified by Cotton-2 for which the desorption yield for 3-nitroaniline-d_4_ is less than 10%, while it is around 100% for bis(2-ethylhexyl) phthalate-d_4_. The compounds in the figure are presented in GC retention order, which means roughly from higher to lower volatility, and there is a clear distinction between quinoline and bis(2-ethylhexyl) phthalate with vapor pressures of 0.06 and 1.4∙10^−7^ mm Hg at 25 °C, respectively (data from PubChem). Still, in spite of the considerably lower volatility of bis(2-ethylhexyl) phthalate, this substance is almost completely desorbed from both cotton materials. This shows that the differences in vapor pressure are not the main reason for these large variations in thermal desorption.

As shown in Fig. 3, Cotton-2 demonstrated the lowest desorption efficiency for all analytes, with the lowest yields for 2-methylbenzothiazole and the nitrogen-containing compounds, here compared to the two isotope-labelled phthalate compounds and benzophenone. The lower yields of the nitrogen-containing compounds indicate a stronger adsorption of these substances to the material via hydrogen bonding and/or polar-polar interactions with the hydroxyl groups on the cellulose surface. This further explains the weaker interaction for more lipophilic compounds, such as bis(2-ethylhexyl) phthalate. These variations in adsorption strength lead to different compound-dependent desorption yields in the ATD. The different desorption efficiencies between the materials may also depend on the physical structure of the cotton fabric, exposing varying amounts of accessible hydroxyl groups on the fiber surfaces. Further, the diffusivity of the cotton materials could also play a role in the thermal desorption. For instance, a less compact material could result in faster diffusion, requiring less time for a complete desorption. The desorption yields for all investigated compounds are presented in Figs. S1–6 in Electronic Supplementary Material.

**Fig. 4:**
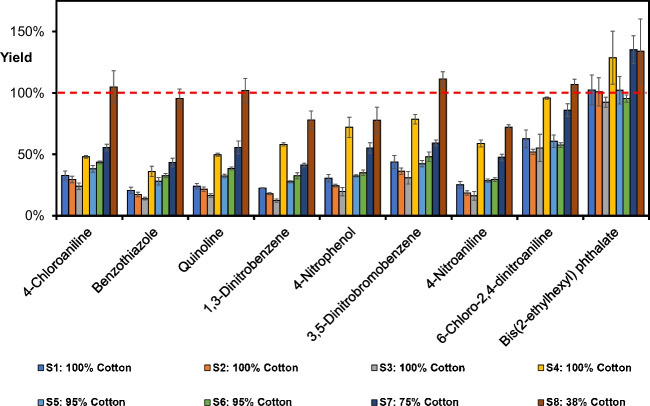
Desorbed amount (%) for selected textile chemicals spiked on eight textile matrices, S1–S8. Samples are from left to right (blue to brown bar) S1 to S8, where S1–S4 contained 100% cotton, S5–S6 95% cotton, S7 75% cotton, and S8 38% cotton. The red dashed line shows 100% yield. The compounds are presented in GC retention order (*n* = 3)

The influence of the cotton material on the ATD desorption yield was further investigated for eight additional textiles, S1–S8 (Table 1). Four textiles, S1–S4, were made of 100% cotton, while S5–S8 were of mixed fabrics containing cotton with various compositions of synthetic fibers. In Fig. 4, the results for some analytes frequently detected as native compounds in everyday garments are shown. The compounds are presented in the diagram in GC retention order.

As for the 100% cotton materials investigated above, these eight garments also showed large differences in the desorption yield. The lowest values were generally found for 95–100% cotton textile materials, with the response for 1,3-dinitrobenzene in textile S3 (100% cotton, gray bar) as low as 12%. The 38% cotton blend, on the other hand, demonstrated generally higher and less varying yields, ranging from 72% to higher. The general conclusion to be drawn is that there are large differences between cotton materials, which must be considered when developing an analytical method based on thermal desorption. The yields for the remaining target compounds are presented in Figs. S7–12.

#### The ATD desorption time and temperature

The ATD desorption time and temperature parameters were investigated for the effect on the desorption yield of spiked analytes, exemplified by the internal standard compounds spiked on Cotton-1 (Table 4). Repeating the 10-min ATD desorption cycle at 175 °C twice resulted in a total desorption time of 30 min at 175 °C. As can be seen in the table, an increased desorption time, including cycles 2 and 3, only resulted in a minor increase of at most 3–4% of the total extracted amount.

**Table 4:** The percentage extracted from spiked Cotton-1 at each 10-min desorption at 175 °C (*n* = 3) followed by 220 °C (*n* = 3)

	Desorption at 175 °C			Continued desorption at 220 °C		
Name	1^st^	2^nd^	3^rd^	4^th^	5^th^	6^th^
Quinoline-d_7_	36 ± 5%	1.4 ± 0.5%	n.d	10 ± 2%	1.5 ± 0.2%	n.d
2-Methylbensothiasole	44 ± 6%	1.4 ± 0.4%	n.d	8 ± 2%	1.2 ± 0.1%	n.d
3-Nitroaniline-d_4_	29 ± 3%	n.d	n.d	n.d	n.d	n.d
Diethyl phthalate-d_4_	81 ± 5%	n.d	n.d	1.7 ± 0.4%	n.d	n.d
4-Nitroaniline-^15^N₂	33 ± 4%	n.d	n.d	n.d	n.d	n.d
Benzophenone-d_10_	75 ± 4%	n.d	n.d	3.1 ± 0.3%	n.d	n.d
2,4-Dinitrobromobenzene-d_3_	57 ± 5%	n.d	n.d	n.d	n.d	n.d
Eicosane-d_42_	100%	n.d	n.d	n.d	n.d	n.d
Bis(2-ethylhexyl) phthalate-d_4_	134 ± 11%	1.3 ± 0.5%	n.d	n.d	n.d	n.d

The percentage relates to the total amount of the extracted compound

*n.d.*, not detected

After these three initial desorption cycles at 175 °C, the desorption temperature was elevated to 220 °C, and three consecutive desorption cycles, cycles 4 to 6, were performed on the same sample. A slight increase in the desorbed amount was observed only for quinoline and 2-methylbenzothiazole, indicating a stronger adsorption of these compounds to the cotton. By increasing the desorption temperature further to 260 °C and 280 °C, the yield from the cotton matrix could be increased for four of the compounds. However, these elevated temperatures cannot be used. Despite a slightly more efficient desorption at these elevated temperatures, the ATD desorption temperature was kept at 175 °C in order to avoid thermal decomposition of any present azo dyes into arylamines, an effect that we have observed earlier [19]. Data on all compounds are given in Tables S6 and S7.

#### Wettability of cotton materials

**Fig. 5:**
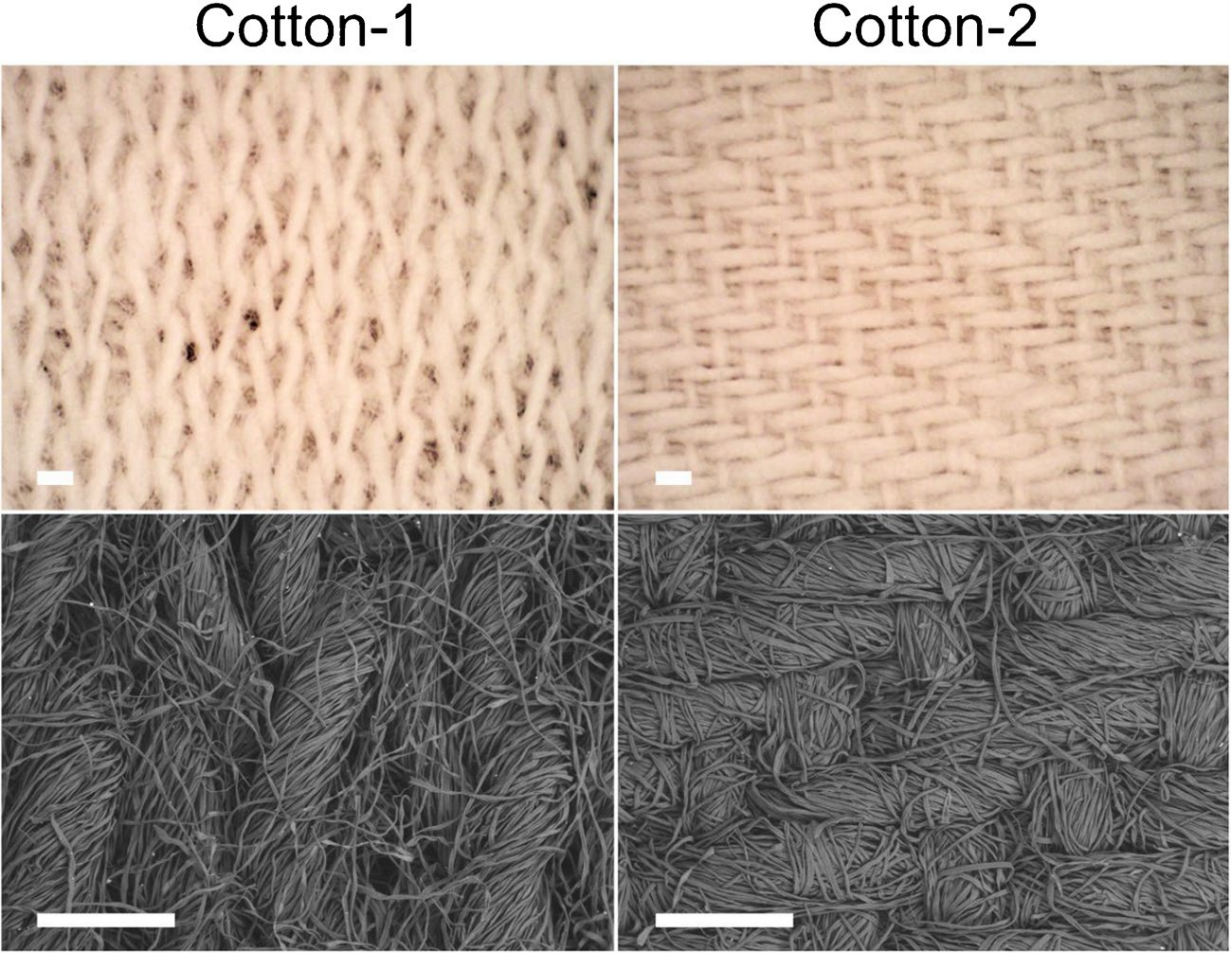
Optical and electronic microscope images of Cotton-1 (left) and Cotton-2 (right). Scale bar: 500 µm

Since both Cotton-1 and Cotton-2 are composed of 100% cellulose and should possess similar surface chemistry, their differences in desorption yields could instead be attributed to physical properties, such as variations in their fabric structure. Microscope images of Cotton-1 and Cotton-2 showed distinct fabric patterns. Cotton-1 is knitted, while Cotton-2 is woven. This implies that they have different thread densities and hence different pore sizes (Fig. 5). This is consistent with their calculated bulk densities (Cotton-1: 252 ± 16 kg/m^3^, Cotton-2: 429 ± 10 kg/m^3^), indicating that Cotton-1 has a larger pore size.

The sorption velocities of liquids with different polarities were measured using a tensiometer. The results are summarized in Table 5.

**Table 5:** Sorption velocities, C values, and water contact angle for Cotton-1 and Cotton-2

	Sorption velocity (× 10^−3^, g^2^/s)					
Material	Hexane	Ethanol	Water	Acetonitrile	C value (mm^5^)	Water contact angle (°)
Cotton-1	128.8 ± 29.5	71.9 ± 8.0	-	71.8 ± 3.2	5.04 ± 1.15	> 90
Cotton-2	78.4 ± 10.9	35.2 ± 3.3	25.6 ± 0.6	154.8 ± 25.4	3.06 ± 0.43	83.9 ± 0.9

For liquids with low polarity and surface tension, such as hexane and ethanol, the sorption velocities were higher and less homogeneous in Cotton-1 than in Cotton-2. This is consistent with their porosities (Cotton-1: 84 ± 1%, Cotton-2: 73 ± 1%). The denser structure of Cotton-2 resulted in a narrower void size distribution, leading to slower and more uniform spreading within the fabric. In contrast, water, a liquid with high polarity and surface tension, did not spontaneously wet Cotton-1, as no sorption of water was detected with the tensiometer, implying a contact angle higher than 90°. This indicates a lower hydrophilicity of Cotton-1 vs Cotton-2. The higher apparent contact angle in Cotton-1 is likely due to water interacting more with air trapped in the pores rather than the fabric itself.

A similar sorption behavior was shown for acetonitrile, resulting in a faster sorption velocity for Cotton-2 than Cotton-1 (Table 5). Although acetonitrile has a lower surface tension than water, both liquids have similar polar to dispersive surface tension ratios (Table 3). This similarity in polarity likely explains why both liquids exhibit comparable wettability trends on the cotton fabrics.

These differences in wettability and sorption behavior may explain the variations in thermal desorption efficiency of spiked analytes between the two fabrics. As acetonitrile penetrates deeper into Cotton-2, it interacts more extensively with its cotton fibers than in Cotton-1, where air pockets might inhibit these interactions. Consequently, the desorption of the analytes is more challenging in Cotton-2 due to stronger interactions with the cotton fibers. The same principle probably applies to many native chemicals introduced into the cotton fibers, especially at the dyeing stage of textile production. The results for Cotton-1 and Cotton-2 align with the other investigated cotton textiles. S4 was the fabric with the highest voids and the highest desorption yields among the spiked textiles of 100% cotton (Fig. 4, Figs. S7–12). Optical and electronic microscope images are to be found in Fig. S13.

### Quantification methods

In the “Examination of parameters affecting the ATD-GC/MS analysis” section, the ATD desorption yield from cotton materials was shown to be strongly dependent on the cotton itself, which can have a strong influence on the accuracy of the quantification. It points out the necessity of a quantification method that can compensate for these effects. The quantification with ATD-GC/MS can be performed using two different strategies: (1) using relative response factors (analyte/IS) calculated from the analysis of a spiked cotton material similar to the sample material, an “External Standard Textile Matrix,” analyzed in a desorption tube separate from the sample, and (2) using only the absolute response factor from internal standard (IS) compounds spiked to the sample, and assuming a response similar to that of the analyte. From this point, the terms “Relative Response Method” and “IS Response Method” are used in the following discussions to refer to quantification using methods 1 and 2, respectively.

#### The “Relative Response Method”

**Fig. 6:**
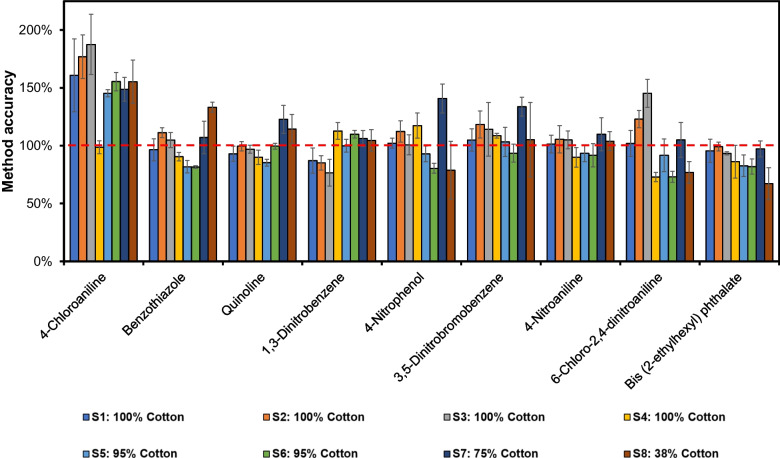
Method accuracy for selected textile chemicals in different matrices calculated using the “Relative Response Method” with Cotton-1 as the “External Standard Textile Matrix.” The red dashed line shows 100% method accuracy. Samples, the colored bars from left to right: S1 to S8. S1–S4: 100% cotton, S5–S6: 95% cotton, S7: 75% cotton, and S8: 38% cotton (*n* = 3)

The “Relative Response Method” was applied by using Cotton-1 as the “External Standard Textile Matrix,” spiked with the standard references and the IS compounds. This was analyzed with the ATD-GC/MS in separate desorption tubes, and the results were used to calculate response factors for the individual analytes towards an appropriately chosen IS component. The reference and the IS compounds were also added to each sample, S1–S8, and the relative response factors were used for the quantification. The results from this “Relative Response Method” are presented in Fig. 6, showing a selected number of compounds frequently occurring in textile garments.

The “Relative Response Method” demonstrated a good accuracy for most compounds, in most cases around 100%. However, of the selected compounds in the figure, 4-chloroaniline was overestimated with as much as 50 to 75% in samples S1–S3 and S5–S8. The IS used for quantification of this analyte was 3-nitroaniline-d₄. A chemically more similar labelled IS as well as more closely eluting would probably improve the accuracy.

**Fig. 7:**
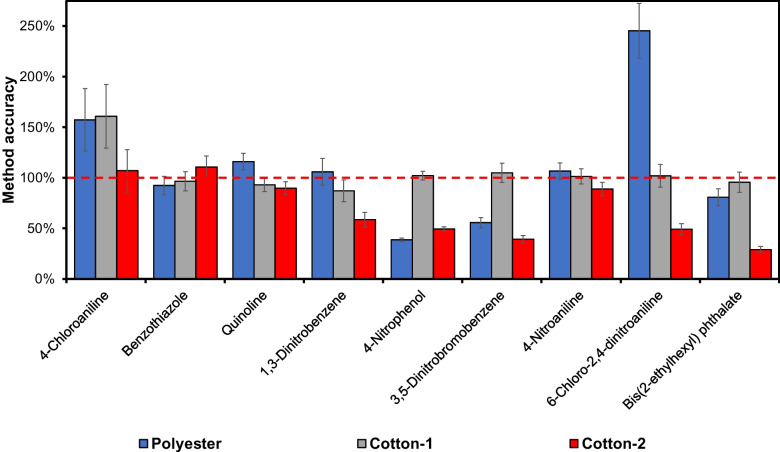
Method accuracy for selected textile chemicals in sample S1 (100% cotton) quantified using the “Relative Response Method” using relative responses analyte/IS from spiked external standard materials (100% polyester, Cotton-1, and Cotton-2). The red dashed line illustrates 100% method accuracy (*n* = 3)

Cotton-2 (also 100% cotton) and 100% polyester, both spiked with reference and IS compounds, were also tested as the “External Standard Textile Matrix” with the “Relative Response Method,” for quantitative analysis of sample S1. In the case of Cotton-2, the influence of the cotton material was revealed. There were now large deviations in the quantifications of the compounds in sample S1 compared to using Cotton-1 as the “External Standard Textile Matrix,” with a strongly decreased accuracy. As an example, there was a decrease in the determined amount of 3,5-dinitrobromobenzene by 60% and for bis(2-ethylhexyl) phthalate by 75% **(**Fig. 7). The use of the 100% polyester textile as the “External Standard Textile Matrix” was not an option either. In this case, the calculated values were between 25 and 240% of the correct amount. This shows a severe drawback with the “Relative Response Method,” the accuracy depends strongly on finding an “External Standard Textile Matrix,” sufficiently similar to the cotton sample matrix to be analyzed. This would make the method very laborious since each sample has to be classified by ATD-GC/MS analysis. Then, a suitable “External Standard Textile Matrix” must be selected in order to obtain the correct relative response factors for the calculations. Results for the remaining analytes are presented in Figs. S14–19.

#### The “IS Response Method”

**Fig. 8:**
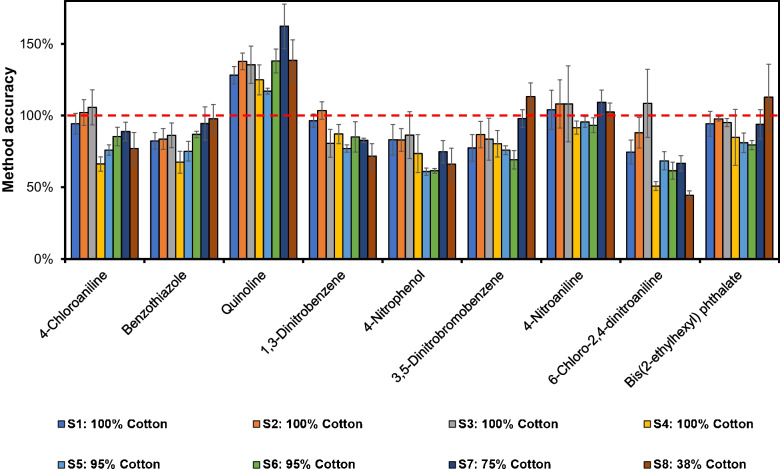
Method accuracy for selected textile chemicals in different textile matrices calculated using the “IS Response Method” (*n* = 3). Samples from left to right: S1 to S8. Samples from left to right: S1 to S8. S1–S4: 100% cotton, S5–S6: 95% cotton, S7: 75% cotton, and S8: 38% cotton. The red dashed line illustrates 100% accuracy

The quantification calculations in the “IS Response Method” utilize the absolute response factor of the internal standard compound, chemically similar to and closest eluting to the analyte in the chromatogram. Figure 8 shows the quantification results for the same spiked samples and the same selected analytes as for the “Relative Response Method” above. The results are mostly between 75 and 100% of the correct value, i.e., the spiked amount. There was an overestimation between 20 and 70% of the amount of quinoline despite using the corresponding deuterium-labelled IS, quinoline-d_7_. The reason for this was interference from the m/z 129 ion from nonanoic acid, which was present in various amounts in all eight samples. In two samples, S4 and S8, 6-chloro-2,4-dinitroaniline showed an accuracy of only around 40% to 50%, respectively. The quantification of this compound used the response for 4-nitroaniline-^15^N_2_ for the calculations. It is likely that a more similar IS compound, however, not available for the present study, would improve the accuracy. The results demonstrate that the absolute response factors from the used IS corrected the differences in thermal desorption efficiencies. To substantially increase the overall accuracy of this “IS Response Method,” a well-composed mix of IS components should be used. The ideal solution would be to use isotope-labelled internal standards for all analytes. Results for the remaining compounds are found in Figs. S20–25.

In summary, when comparing the results for the “Relative Response Method” with the “IS Response Method,” the latter method gives comparably good quantification results while correcting for the different cotton matrices of the samples. Conversely, the former method strongly depends on finding a suitable “External Standard Textile Matrix” to calculate accurate relative response factors. This means substantially more work is needed to identify the character of the cotton sample, find a suitable standard textile, and include several runs with this external standard in an analysis series. In the “IS Response Method,” good statistics for the absolute response factors are obtained, requiring only the results from the samples. n-Eicosane-d_42_ should be added to each sample as a volumetric standard to control the system’s repeatability.

### ATD-GC/MS for cotton analysis

#### ATD-GC/MS analysis of textile samples—comparison with solvent extraction

To further evaluate the two quantification strategies applied for the online ATD-GC/MS method, they were compared with solvent extraction combined with offline GC/MS analysis (SE-GC/MS). Four cotton textiles were selected for analysis, Textile-1 to Textile-4 (Table 2). These samples were selected since they contain several compounds frequently found in textiles. All quantitative data are presented in Table S8. In Electronic Supplementary Material, ATD-GC/MS chromatograms of a standard solution on 100% cotton and polyester, respectively, are illustrated in Figs. S26a and b.

**Fig. 9:**
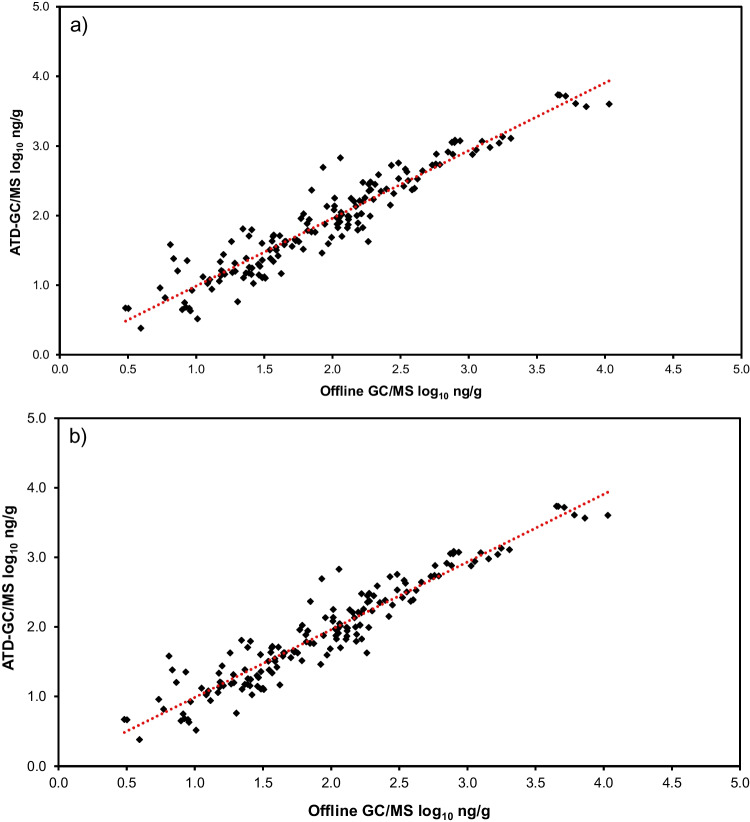
**a** Determined concentrations using SE-GC/MS analysis (x-axis) vs ATD-GC/MS (y-axis) using the “Relative Response Method” (Slope = 0.99, R^2^ = 0.86, RMSE = 28%, upper diagram). **b** Determined concentrations using SE-GC/MS analysis (x-axis) and ATD-GC/MS (y-axis) using the “IS Response Method” (Slope = 0.97, R^2^ = 0.90, RMSE = 24%, lower diagram)

In Fig. 9a, the ^10^log concentrations obtained for the four samples analyzed with online ATD-GC/MS using the “Relative Response Method” for quantification, with Cotton-1 as the “External Standard Textile Matrix,” is plotted against the corresponding results obtained by SE-GC/MS. In Fig. 9b, the same kind of plot is shown but for the “IS Response Method.” As can be seen, both quantification methods for the ATD-GC/MS analysis agree rather well with the corresponding SE-GC/MS analysis. As expected from the results described above, the “Relative Response Method” gave a somewhat higher agreement with the SE-GC/MS method (Slope = 0.99) compared to the “IS Response Method” (Slope = 0.97, Fig. 9b). In both cases, the R^2^ values were similar.

Altogether, the results suggest that using the “IS Response Method” would give sufficient accuracy and precision for samples independent of the investigated cotton fabric. This method would most likely be the best option for large-scale quantitative screenings due to (1) the relatively small differences in method accuracy compared to the use of the “Relative Response Method” on a well-defined matrix and (2) a more straightforward workflow with no need for choosing and evaluating a representative “External Standard Textile Matrix,” and (3) eliminating the need for several external calibrations in between sample runs. Labelled and closely eluting IS compounds are recommended to obtain the maximum accuracy for the “IS Response Method.”

#### Linearity and detection limits

The responses for all the tested compounds were found to be linear within the investigated range, i.e., approximately 1 to 100 μg/g textile, with analytes spiked on a 20 mg textile matrix. The correlation coefficients (R^2^) for cotton for all calibration curves were 0.92 to 0.99. The exception was bis(2-ethylhexyl) phthalate (R^2^ = 0.85). The deviation for this analyte was found to be due to contamination from the laboratory environment (Table S9).

The instrumental LODs, tested by spiking with amounts close to the detection limits on a textile free from the investigated chemicals, were in the range of 0.1–8 ng for polyester and 0.1–40 ng for cotton. The latter is due to cotton’s lower thermal desorption efficiency compared to polyester. The MDL values for the ATD-GC/MS method using around 20 mg of textile were, for most investigated compounds, well below one µg/g textile. Several of the investigated target analytes were arylamines, for which the MDL was less than 0.030 µg/g textile. The EU REACH regulation allows a maximum level of 30 µg/g textile for individual arylamines, a value more than 100 times higher than the MDL achieved with ATD-GC/MS. Information for the individual analytes is found in Table S10.

## Conclusions

Together with our previous study, the present results show the applicability of ATD-GC/MS for quantitative analysis of at least 75% of all textile materials on the clothing retail market, comprising polyester, polyester blends, cotton, and cotton blends.

Cotton shows differences in thermal desorption efficiencies depending on the type of fabric. Our results show that two different cotton fabrics can have different water contact angles, i.e., different surface energies primarily due to their fabric density and porosity. Nevertheless, our proposed quantitative ATD-GC/MS method can overcome these variations.

ATD-GC/MS enabled accurate quantification of several classes of textile chemicals, with method detection limits well below current EU regulations for quinoline, arylamines, and phthalates. Based on the results from the present study, a simplified workflow is suggested, using absolute responses from well-selected internal standards for quantification. This procedure does not require selecting and validating an external standard textile matrix or external calibrations.

Due to the fast fashion trends, the number of substances and the complexity of the chemical residues in everyday garments can be expected to continue increasing. By applying ATD-GC/MS with a non-targeted approach, the detection and identification of new emerging, possibly mutagenic or skin-sensitizing compounds can be realized.

The performance of ATD-GC/MS for other types of textiles, such as viscose and other artificial cellulosic fibers, remains to be investigated in future work.

## Supplementary Information

Below is the link to the electronic supplementary material.

ESM 1 (PDF 5.25 MB)

## Data Availability

Data will be made available upon request.
