# Supplementary material for: Automated thermal desorption-gas chromatography/mass spectrometry for screening of hazardous chemicals in cotton and cotton blend garments—analytical challenges
Source: Anal Bioanal Chem. 2025 Jul 8;417(21):4775–89. doi: 10.1007/s00216-025-05993-y (PMC12367912; doi:10.1007/s00216-025-05993-y)
Supplement: Supplementary file 1 — (PDF 5.25 MB) [file 216_2025_5993_MOESM1_ESM.pdf]

Electronic Supplementary Material

Automated thermal desorption-gas chromatography/mass spectrometry for screening of hazardous chemicals in cotton and cotton blend garments – analytical challenges

Tim Åström, Conny Östman, Ioannis Sadiktsis, and Maria-Ximena Ruiz Caldas, Ulrika Nilsson*

Department of Chemistry, Stockholm University

SE-106 91 Stockholm, Sweden

Correspondence:

*) Ulrika Nilsson, Dep of Chemistry, Stockholm University, SE-106 91 Stockholm, Sweden.

e-mail: unils@su.se

Table S1: Reference compounds used in the present study

Compounds	CAS	Molecular formula	Molecular mass (Da)	log P*	Purity	Supplier
**Internal Standards (IS)**						
Quinoline-d_7	34071-94-8	C_9D_7N	136	2.0	98%	Cambridge Isotopes, Tewksbury, USA
2-Methylbenzothiazole	120-75-2	C_8H_7NS	149	3.0	99%	Sigma Aldrich, St Louis, USA
3-Nitroaniline-d_4	115044-52-5	$C_6D_4H_2N_2O_2$	142	1.4	98.7%	Cambridge Isotopes, Tewksbury, USA
Diethyl phthalate-d_4	93952-12-6	$C_{12}D_4H_{10}O_4$	226	2.5	98%	Cambridge Isotopes, Tewksbury, USA
4-Nitroaniline-$^{15}N_2$	119516-81-3	$C_6H_6N_2O_2$	140	1.4	98%	Sigma Aldrich, St Louis, USA
Benzophenone-d_{10}	22583-75-1	$C_{13}D_{10}O$	192	3.4	99%	Sigma Aldrich, St Louis, USA
2,4-Dinitrobromobenzene-d_3	1313734-81-4	$C_6H_3BrN_2O_4$	250	2.2	98%	Sigma Aldrich, St Louis, USA
Bis(2-ethylhexyl) phthalate-d_4	93951-87-2	$C_{24}D_4H_{34}O_4$	394	7.4	98%	Cambridge Isotopes, Tewksbury, USA
n-Eicosane-d_{42}	62369-67-9	$C_{20}D_{42}$	324	10.4	98%	Sigma Aldrich, St Louis, USA
**Quinolines**						
Quinoline	91-22-5	C_9H_7N	129	2.0	97%	Merck, Darmstadt, Germany
Isoquinoline	119-65-3	C_9H_7N	129	2.1	97%	Sigma Aldrich, St Louis, USA
2-Methylquinoline	91-63-4	$C_{10}H_9N$	143	2.6	95%	Sigma Aldrich, St Louis, USA
8-Methylquinoline	611-32-5	$C_{10}H_9N$	143	2.6	97%	Sigma Aldrich, St Louis, USA
6-Methylquinoline	91-62-3	$C_{10}H_9N$	143	2.6	N.S.	Labkemi AB, Stockholm, Sweden
3-Methylquinoline	612-58-8	$C_{10}H_9N$	143	2.5	99%	Sigma Aldrich, St Louis, USA
4-Methylquinoline	491-35-0	$C_{10}H_9N$	143	2.6	99%	Sigma Aldrich, St Louis, USA
2,6-Dimethylquinoline	877-43-0	$C_{11}H_{11}N$	157	3.0	N.S.	K&K laboratories, Cleveland, Ohio, USA
2,4-Dimethylquinoline	1198-37-4	$C_{11}H_{11}N$	157	3.0	N.S.	K&K laboratories, Cleveland, Ohio, USA
**Arylamines**						
4-Chloroaniline	106-47-8	C_6H_6ClN	127	1.9	98%	Sigma Aldrich, St Louis, USA
2-Bromoaniline	615-36-1	C_6H_6BrN	171	2.1	98%	Sigma Aldrich, St Louis, USA
3-Bromoaniline	591-19-5	C_6H_6BrN	171	2.1	98%	Sigma Aldrich, St Louis, USA
4-Bromoaniline	106-40-1	C_6H_6BrN	171	2.3	97%	Sigma Aldrich, St Louis, USA
2-Nitroaniline	88-74-4	$C_6H_6N_2O_2$	138	1.9	98%	Sigma Aldrich, St Louis, USA
3,4-Dichloroaniline	95-76-1	$C_6H_5Cl_2N$	161	2.7	98%	Sigma Aldrich, St Louis, USA

3-Nitroaniline	99-09-2	$C_6H_6N_2O_2$	138	1.4	98%	Sigma Aldrich, St Louis, USA
2,6-dichloro-1,4-phenylenediamine	609-20-1	$C_6H_6Cl_2N_2$	176	2.4	90%	Apollo Scientific, Stockport, UK
4-Chloro-2-nitroaniline	89-63-4	$C_6H_5ClN_2O_2$	172	2.7	99%	Sigma Aldrich, St Louis, USA
4-Nitroaniline	100-01-6	$C_6H_6N_2O_2$	138	1.4	99%	Sigma Aldrich, St Louis, USA
2-Chloro-4-nitroaniline	121-87-9	$C_6H_5ClN_2O_2$	172	2.1	99%	Sigma Aldrich, St Louis, USA
2,6-Dichloro-4-nitroaniline	99-30-9	$C_6H_4Cl_2N_2O_2$	206	2.9	96%	Sigma Aldrich, St Louis, USA
Dibenzylamine	103-49-1	$C_{14}H_{15}N$	197	2.7	97%	Sigma Aldrich, St Louis, USA
3,5-Dibromo-1,2-phenylenediamine	1575-38-8	$C_6H_6Br_2N_2$	266	1.9	95%	Enamine Ltd, Kyiv, Ukraine
2-Chloro-4,6-dinitroaniline	3531-19-9	$C_6H_4ClN_3O_4$	217	2.1	97%	Sigma Aldrich, St Louis, USA
2,6-Dibromo-4-nitroaniline	827-94-1	$C_6H_4Br_2N_2O_2$	266	3.2	97%	Sigma Aldrich, St Louis, USA
2,4-Dinitroaniline	97-02-9	$C_6H_5N_3O_4$	183	1.8	98%	Sigma Aldrich, St Louis, USA
2-Bromo-4,6-dinitroaniline	1817-73-8	$C_6H_4BrN_3O_4$	263	2.1	94%	Sigma Aldrich, St Louis, USA
**Nitrobenzene compounds**						
3,5-Dichloronitrobenzene	618-62-2	$C_6H_3Cl_2NO_2$	192	3.1	95%	Sigma Aldrich, St Louis, USA
1,4-Dinitrobenzene	100-25-4	$C_6H_4N_2O_4$	168	1.5	98%	Sigma Aldrich, St Louis, USA
1,3-Dinitrobenzene	99-65-0	$C_6H_4N_2O_4$	168	1.5	A.S.	Sigma Aldrich, St Louis, USA
1,2-Dinitrobenzene	528-29-0	$C_6H_4N_2O_4$	168	1.7	97%	Sigma Aldrich, St Louis, USA
2,4-Dinitrochlorobenzene	97-00-7	$C_6H_3ClN_2O_4$	202	2.3	99%	Sigma Aldrich, St Louis, USA
2,5-Dinitrochlorobenzene	619-16-9	$C_6H_3ClN_2O_4$	202	2.1	N.S.	Toronto Research Chemicals, Toronto, CA
3,5-Dinitrobromobenzene	18242-39-2	$C_6H_3BrN_2O_4$	247	2.0	N.S.	Apollo Scientific, Stockport, UK
**Phthalates**						
Dimethyl phthalate	131-11-3	$C_{10}H_{10}O_4$	194	1.6	99%	Sigma Aldrich, St Louis, USA
Dimethyl terephthalate	120-61-6	$C_{10}H_{10}O_4$	194	2.2	99%	Sigma Aldrich, St Louis, USA
Dipropyl phthalate	131-16-8	$C_{14}H_{18}O_4$	250	4.0	98%	Sigma Aldrich, St Louis, USA
Isobutyl phthalate	84-69-5	$C_{16}H_{22}O_4$	278	4.1	99%	Sigma Aldrich, St Louis, USA
Benzyl butyl phthalate	85-68-7	$C_{19}H_{20}O_4$	312	4.9	98%	Sigma Aldrich, St Louis, USA
Bis(2-ethylhexyl) phthalate	117-81-7	$C_{24}H_{38}O_4$	390	7.4	99.5%	Sigma Aldrich, St Louis, USA
Dioctyl phthalate	117-84-0	$C_{24}H_{38}O_4$	390	9.1	A.S.	Sigma Aldrich, St Louis, USA
Bis(2-ethylhexyl) terephthalate	6422-86-2	$C_{24}H_{38}O_4$	390	7.4	A.S.	Sigma Aldrich, St Louis, USA
**Additional compounds**						

Naphthalene	91-20-3	$C_{10}H_8$	128	3.3	A.S.	Honeywell, Charlotte, USA
2-Phenoxyethanol	122-99-6	$C_8H_{10}O_2$	138	1.2	99%	Sigma Aldrich, St Louis, USA
Benzothiazole	95-16-9	C_7H_5NS	135	2.1	96%	Sigma Aldrich, St Louis, USA
Biphenyl	92-52-4	$C_{12}H_{10}$	154	4.0	99%	Sigma Aldrich, St Louis, USA
3-Nitrophenol	554-84-7	$C_6H_5NO_3$	139	2.0	99%	Sigma Aldrich, St Louis, USA
4-Nitrophenol	100-02-7	$C_6H_5NO_3$	139	1.9	99%	Sigma Aldrich, St Louis, USA
2,4-Dinitrophenol	51-28-5	$C_6H_4N_2O_5$	184	1.7	A.S.	Sigma Aldrich, St Louis, USA
Tributyl phosphate	126-73-8	$C_{12}H_{27}O_4P$	266	2.9	97%	Sigma Aldrich, St Louis, USA
Diethyl benzyl phosphonate	1080-32-6	$C_{11}H_{17}O_3P$	228	1.7	99%	Sigma Aldrich, St Louis, USA
Benzophenone	119-61-9	$C_{13}H_{10}O$	182	3.4	A.S.	Sigma Aldrich, St Louis, USA
2-Hydroxybenzothiazole	934-34-9	C_7H_5NOS	151	1.8	98%	Sigma Aldrich, St Louis, USA
Benzyl benzoate	120-51-4	$C_{14}H_{12}O_2$	212	4.0	99%	Sigma Aldrich, St Louis, USA
Phenanthrene	85-01-8	$C_{14}H_{10}$	178	4.5	98%	Sigma Aldrich, St Louis, USA
Anthracene	120-12-7	$C_{14}H_{10}$	178	4.4	97%	Sigma Aldrich, St Louis, USA
Tris(2-ethylhexyl) phosphate	78-42-2	$C_{24}H_{51}O_4P$	434	8.9	97%	Sigma Aldrich, St Louis, USA
Triphenyl phosphate	115-86-6	$C_{18}H_{15}O_4P$	326	4.6	99%	Sigma Aldrich, St Louis, USA

Table S2: Selected ions and measured m/z for studied compounds

Normalized to IS	Internal standards	RT (min)	Target ion (Q1)	Qualifier ions, q1/q2	Dwell time (ms)
**Internal standards**					
	Quinoline-d_7	6.3	136	108 / 80	15
	2-Methylbenzothiazole	7.0	149	108 / 69	10
	3-Nitroaniline-d_4	10.1	142	96 / 69	15
	Diethylphthalate-d_4	10.6	153	181 / 80	10
**Eicosane-d_{42}**	4-Nitroaniline-$^{15}N_2$	11.3	140	192 / 82	10
	Benzophenone-d_{10}	11.9	110	109 / 93	10
	2,4-Dinitrobromobenzene-d_3	12.4	249	250 / 157	10
	Bis (2-ethylhexyl) Phthalate-d_4	18.9	153	283 / 171	15
	Eicosane-d_{42}	12.1	66	98 / 82	10
**Quinolines**					
	Quinoline	6.4	129	102 / 76	15
	Isoquinoline	6.7	129	102 / 75	15
	2-Methylquinoline	7.1	143	128 / 115	10
	8-Methylquinoline	7.2	143	115 / 89	10
**Quinoline-d7**	6-Methylquinoline	7.8	143	115 / 89	15
	3-Methylquinoline	7.9	143	115 / 89	15
	4-Methylquinoline	8.2	143	115 / 89	15
	2,6-Dimethylquinoline	8.5	157	142 / 115	15
	2,4-Dimethylquinoline	8.9	157	142 / 115	15
**Arylamines**					
	4-Chloroaniline	5.9	127	92 / 65	15
	2-Bromoaniline	6.0	171	92 / 65	15
	3-Bromoaniline	7.2	171	92 / 65	10
**3-Nitroaniline-d_4**	4-Bromoaniline	7.3	171	92 / 65	10
	2-Nitroaniline	9.0	138	92 / 65	15
	3,4-Dichloroaniline	9.0	161	163 / 126	15
	3-Nitroaniline	10.2	138	92 / 65	15
	2,6-Dichloro-1,4-phenylenediamine	11.3	176	114 / 78	10
	4-Chloro-2-nitroaniline	11.4	172	126 / 99	10
	4-Nitroaniline	11.9	138	108 / 92	10
	2-Chloro-4-nitroaniline	12.8	172	174 / 142	15
	2,6-Dichloro-4-nitroaniline	12.8	206	176 / 124	15
**4-Nitroaniline-$^{15}N_2$**	Dibenzylamine	12.1	106	196 / 91	15
	3,5-Dibromobenzene-1,2-diamine	14.0	266	268 / 185	15
	6-Chloro-2,4-dinitroaniline	15.3	217	187 / 125	15
	2,6-Dibromo-4-nitroaniline	15.3	266	296 / 170	15
	2,4-Dinitroaniline	16.0	183	153 / 91	15
	2-Bromo-4,6-dinitroaniline	16.5	261	231 / 169	15
**Nitrobenzenes**					
**2,4-Dinitrobromobenzene-d_3**	3,5-Dichloronitrobenzene	7.4	145	191 / 109	10
	1,4-Dinitrobenzene	9.7	168	122 / 92	15

	1,3-Dinitrobenzene	9.8	168	122 / 92	15
	1,2-Dinitrobenzene	10.1	168	76 / 63	15
	2,4-Dinitrochlorobenzene	11.2	202	110 / 75	10
	2,5-Dinitrochlorobenzene	11.0	202	110 / 75	10
	3,5-Dinitrobromobenzene	11.9	246	248 / 155	10
**Phthalates**					
**Diethylphthalate-d_4**	Dimethyl phthalate	9.2	163	194 / 77	15
	Dimethyl terephthalate	9.4	163	194 / 135	15
	Dipropyl phthalate	12.5	149	104 / 76	15
	Isobutyl phthalate	13.2	149	223 / 104	15
**Bis(2-ethylhexyl) phthalate-d_4**	Benzyl butyl phthalate	18.5	149	206 / 104	15
	Bis(2-ethylhexyl) phthalate	18.9	149	279 / 167	15
	Dioctyl phthalate	20.1	149	279 / 71	15
	Bis(2-ethylhexyl) terephthalate	20.5	149	261 / 167	15
**Additional compounds**					
**Quinoline-d_7**	Naphthalene	5.3	128	102 / 51	15
	2-Phenoxyethanol	5.9	94	138 / 77	15
**2-Methylbensothiasole**	Benzothiazole	6.3	135	108 / 69	15
**Benzophenone-d_{10}**	Biphenyl	7.8	154	115 / 76	15
**3-Nitroaniline-d_4**	3-Nitrophenol	9.3	139	93 / 65	15
	4-Nitrophenol	10.2	139	109 / 81	15
	2,4-Dinitrophenol	9.7	184	154 / 107	15
**Benzophenone-d_{10}**	Tributyl phosphate	10.4	99	211 / 155	10
	Diethylbenzylphosphonate	10.5	228	- / -	10
	Benzophenone	11.4	105	182 / 77	10
	2-Hydroxybenzothiazole	12.5	151	123 / 96	10
**Diethylphthalate-d_4**	Benzyl benzoate	12.8	105	212 / 77	15
**Bis(2-ethylhexyl) phthalate-d_4**	Tris(2-ethylhexyl) phosphate	17.6	99	113 / 57	15
	Triphenyl phosphate	19.2	325	326 / 215	15

Table S3: Matrix-spike experiments and offline GC/MS

**Compounds**	**RT (min)**	**Absolute recovery (%)**	**Corrected recovery (%)**
**Quinolines**			
Quinoline-d_7	5.6	21 ± 2%	88 ± 9%
Quinoline	5.6	18 ± 2%	96 ± 1%
Isoquinoline	5.9	18 ± 2%	99 ± 1%
2-Methylquinoline	6.4	20 ± 2%	98 ± 2%
8-Methylquinoline	6.5	23 ± 2%	98 ± 3%
6-Methylquinoline	7.0	20 ± 1%	102 ± 4%
3-Methylquinoline	7.1	20 ± 1%	100 ± 3%
4-Methylquinoline	7.4	21 ± 1%	102 ± 4%
2,6-Dimethylquinoline	7.7	23 ± 1%	102 ± 4%
2,4-Dimethylquinoline	8.1	26 ± 1%	99 ± 4%
**Arylamines**			
3-Nitroaniline-d_4	9.4	22 ± 1%	91 ± 5%
4-Nitroaniline-$^{15}N_2$	11.1	20 ± 1%	92 ± 5%
4-Chloroaniline	5.2	19 ± 2%	105 ± 3%
2-Bromoaniline	5.3	22 ± 2%	94 ± 1%
3-Bromoaniline	6.5	24 ± 2%	101 ± 3%
4-Bromoaniline	6.5	23 ± 2%	103 ± 3%
2-Nitroaniline	8.2	22 ± 1%	94 ± 1%
3,4-Dichloroaniline	8.3	27 ± 2%	106 ± 4%
3-Nitroaniline	9.4	22 ± 1%	100 ± 1%
2,6-Dichloro-1,4-phenylenediamine	10.5	12 ± 1%	77 ± 2%
4-Chloro-2-nitroaniline	10.6	24 ± 1%	99 ± 1%
4-Nitroaniline	11.1	19 ± 1%	99 ± 1%
Dibenzylamine	11.3	23 ± 2%	80 ± 4%
2-Chloro-4-nitroaniline	12.0	22 ± 1%	99 ± 1%
2,6-Dichloro-4-nitroaniline	12.0	27 ± 2%	103 ± 2%
3,5-Dibromo-2-phenylenediamine	13.2	8 ± 1%	116 ± 14%
6-Chloro-2,4-dinitroaniline	14.5	16 ± 1%	114 ± 3%
2,6-Dibromo-4-nitroaniline	14.4	31 ± 2%	110 ± 2%
2,4-Dinitroaniline	15.2	9 ± 1%	129 ± 7%
2-Bromo-4,6-dinitroaniline	15.6	18 ± 2%	116 ± 4%
**Nitrobenzenes**			
2,4-Dinitrobromobenzene-d_3	11.6	25 ± 1%	83 ± 2%
3,5-Dichloronitrobenzene	6.6	27 ± 2%	107 ± 4%
1,4-Dinitrobenzene	8.9	18 ± 1%	104 ± 4%
1,3-Dinitrobenzene	9.0	20 ± 1%	105 ± 4%
1,2-Dinitrobenzene	9.3	25 ± 1%	107 ± 3%
2,5-Dinitrochlorobenzene	10.2	22 ± 2%	107 ± 5%
2,4-Dinitrochlorobenzene	10.4	24 ± 2%	104 ± 5%
3,5-Dinitrobromobenzene	11.1	23 ± 2%	114 ± 6%
**Phthalates**			
Diethylphthalate-d_4	9.8	44 ± 1%	88 ± 2%

Bis(2-ethylhexyl) phthalate-d_4	18.1	85 ± 1%	99 ± 2%
Dimethyl phthalate	8.4	29 ± 2%	103 ± 4%
Dimethyl terephthalate	8.7	23 ± 1%	100 ± 4%
Dipropyl phthalate	11.7	55 ± 3%	110 ± 4%
Isobutyl phthalate	12.4	60 ± 2%	118 ± 7%
Benzyl butyl phthalate	17.6	62 ± 3%	98 ± 4%
Bis(2-ethylhexyl) phthalate	18.1	73 ± 32%	164 ± 48%
Dioctyl phthalate	19.3	81 ± 2%	101 ± 1%
Bis(2-ethylhexyl) terephthalate	19.7	77 ± 2%	102 ± 1%
**Additional compounds**			
2-Methylbenzothiazole	6.2	22 ± 2%	90 ± 9%
Benzophenone-d_{10}	10.5	35 ± 1%	89 ± 3%
Naphthalene	4.7	26 ± 6%	97 ± 13%
2-Phenoxyethanol	5.2	21 ± 2%	96 ± 2%
Benzothiazole	5.6	19 ± 2%	95 ± 1%
Biphenyl	7.1	36 ± 2%	101 ± 3%
Tributyl phosphate	9.7	64 ± 2%	111 ± 2%
Benzophenone	10.6	34 ± 2%	105 ± 3%
Benzyl benzoate	12.0	42 ± 2%	108 ± 4%
Tris(2-ethylhexyl) phosphate	16.8	79 ± 1%	101 ± 1%
Triphenyl phosphate	18.4	58 ± 3%	98 ± 3%

22 Table S4: Stability of target compounds for ATD-GC/MS

Compounds	STD1-3: 6-h	STD4-6: 9-h	STD7-9: 12-h	STD10-12: 15-h	STD13-15: 18-h	STD16-18: 21-h	STD19-21: 24-h	STD22-24: 27-h
**Quinolines**								
Quinoline	103 ± 8%	98 ± 7%	103 ± 7%	96 ± 2%	102 ± 5%	105 ± 6%	97 ± 5%	83 ± 11%
Isoquinoline	94 ± 4%	97 ± 8%	105 ± 7%	95 ± 13%	106 ± 9%	105 ± 10%	102 ± 8%	87 ± 13%
2-Methylquinoline	96 ± 5%	99 ± 10%	101 ± 11%	94 ± 7%	101 ± 6%	110 ± 3%	98 ± 7%	92 ± 10%
8-Methylquinoline	103 ± 1%	104 ± 8%	106 ± 13%	88 ± 10%	102 ± 10%	102 ± 4%	100 ± 9%	82 ± 16%
6-Methylquinoline	101 ± 8%	103 ± 5%	101 ± 13%	94 ± 8%	103 ± 8%	108 ± 8%	94 ± 5%	90 ± 12%
3-Methylquinoline	94 ± 3%	101 ± 4%	102 ± 14%	100 ± 4%	106 ± 3%	107 ± 7%	90 ± 6%	92 ± 14%
4-Methylquinoline	97 ± 10%	102 ± 9%	101 ± 8%	100 ± 16%	105 ± 7%	107 ± 2%	95 ± 4%	88 ± 6%
2,6-Dimethylquinoline	98 ± 6%	99 ± 14%	103 ± 19%	91 ± 8%	108 ± 8%	107 ± 13%	93 ± 7%	90 ± 10%
2,4-Dimethylquinoline	97 ± 12%	105 ± 17%	102 ± 13%	87 ± 9%	100 ± 8%	108 ± 4%	102 ± 11%	92 ± 8%
**Arylamines**								
4-Chloroaniline	103 ± 9%	103 ± 4%	106 ± 9%	91 ± 15%	104 ± 5%	100 ± 1%	98 ± 7%	86 ± 10%
2-Bromoaniline	115 ± 8%	103 ± 11%	106 ± 17%	83 ± 5%	103 ± 13%	102 ± 2%	93 ± 4%	79 ± 13%
3-Bromoaniline	100 ± 5%	109 ± 4%	99 ± 14%	89 ± 4%	101 ± 8%	109 ± 6%	97 ± 5%	85 ± 5%
4-Bromoaniline	104 ± 3%	110 ± 11%	104 ± 18%	87 ± 18%	98 ± 9%	99 ± 3%	100 ± 13%	86 ± 9%
2-Nitroaniline	101 ± 13%	89 ± 5%	100 ± 16%	101 ± 15%	105 ± 12%	106 ± 4%	110 ± 10%	85 ± 6%
3,4-Dichloroaniline	105 ± 14%	103 ± 13%	103 ± 18%	86 ± 10%	100 ± 5%	104 ± 7%	92 ± 5%	90 ± 8%
3-Nitroaniline	99 ± 3%	94 ± 8%	103 ± 22%	101 ± 14%	103 ± 6%	108 ± 12%	101 ± 7%	88 ± 11%
2,6-Dichloro-1,4-phenylenediamine	113 ± 33%	120 ± 22%	94 ± 34%	89 ± 22%	78 ± 15%	116 ± 2%	95 ± 21%	69 ± 4%
4-Chloro-2-nitroaniline	95 ± 5%	93 ± 9%	104 ± 12%	96 ± 7%	101 ± 4%	101 ± 5%	101 ± 6%	94 ± 7%
4-Nitroaniline	97 ± 2%	100 ± 10%	100 ± 19%	95 ± 4%	98 ± 4%	110 ± 3%	96 ± 7%	94 ± 6%
2-Chloro-4-nitroaniline	96 ± 6%	95 ± 15%	98 ± 17%	93 ± 3%	103 ± 6%	105 ± 7%	101 ± 4%	100 ± 9%
2,6-Dichloro-4-nitroaniline	90 ± 4%	88 ± 18%	107 ± 16%	89 ± 8%	102 ± 6%	107 ± 7%	108 ± 5%	98 ± 13%
Dibenzylamine	82 ± 6%	80 ± 22%	109 ± 19%	80 ± 6%	101 ± 13%	111 ± 11%	123 ± 12%	105 ± 16%
6-Chloro-2,4-dinitroaniline	91 ± 3%	89 ± 20%	99 ± 27%	90 ± 7%	107 ± 10%	112 ± 14%	112 ± 8%	103 ± 14%
2,6-Dibromo-4-nitroaniline	87 ± 4%	85 ± 18%	103 ± 28%	85 ± 7%	105 ± 10%	113 ± 16%	118 ± 7%	104 ± 13%
2,4-Dinitroaniline	93 ± 1%	94 ± 23%	99 ± 26%	96 ± 1%	110 ± 7%	103 ± 3%	105 ± 6%	103 ± 14%

2-Bromo-4,6-dinitroaniline	88 ± 2%	89 ± 20%	102 ± 26%	92 ± 5%	106 ± 9%	113 ± 8%	110 ± 7%	102 ± 12%
**Nitrobenzene compounds**								
3,5-Dichloronitrobenzene	134 ± 3%	109 ± 8%	124 ± 28%	79 ± 12%	100 ± 10%	90 ± 8%	89 ± 5%	68 ± 15%
1,4-Dinitrobenzene	109 ± 7%	112 ± 19%	99 ± 19%	98 ± 11%	102 ± 11%	95 ± 6%	92 ± 6%	89 ± 15%
1,3-Dinitrobenzene	117 ± 22%	125 ± 13%	108 ± 25%	116 ± 19%	118 ± 13%	101 ± 4%	107 ± 7%	96 ± 12%
1,2-Dinitrobenzene	110 ± 9%	104 ± 5%	96 ± 18%	91 ± 18%	101 ± 5%	106 ± 6%	96 ± 2%	89 ± 10%
2,5-Dinitrochlorobenzene	100 ± 12%	105 ± 6%	100 ± 24%	97 ± 15%	108 ± 5%	97 ± 3%	100 ± 8%	85 ± 6%
2,4-Dinitrochlorobenzene	102 ± 13%	106 ± 11%	102 ± 23%	98 ± 10%	104 ± 3%	96 ± 3%	94 ± 1%	88 ± 10%
3,5-Dinitrobromobenzene	96 ± 7%	95 ± 8%	102 ± 18%	93 ± 13%	106 ± 6%	102 ± 6%	104 ± 4%	95 ± 11%
**Phthalates**								
Dimethyl phthalate	106 ± 9%	110 ± 5%	100 ± 18%	101 ± 13%	100 ± 7%	97 ± 3%	89 ± 1%	85 ± 6%
Dimethyl terephthalate	115 ± 6%	121 ± 19%	94 ± 21%	105 ± 11%	99 ± 25%	93 ± 7%	81 ± 8%	78 ± 8%
Dipropyl phthalate	90 ± 4%	92 ± 3%	105 ± 13%	88 ± 12%	104 ± 7%	105 ± 1%	108 ± 1%	94 ± 9%
Isobutyl phthalate	91 ± 5%	92 ± 5%	102 ± 16%	87 ± 13%	106 ± 10%	104 ± 2%	113 ± 1%	97 ± 10%
Benzyl butyl phthalate	86 ± 4%	82 ± 9%	104 ± 22%	87 ± 12%	108 ± 12%	106 ± 2%	123 ± 1%	108 ± 10%
Bis(2-ethylhexyl) phthalate	94 ± 23%	80 ± 28%	114 ± 20%	113 ± 21%	109 ± 22%	102 ± 10%	92 ± 20%	104 ± 12%
Dioctyl phthalate	87 ± 3%	82 ± 10%	106 ± 21%	85 ± 11%	107 ± 15%	102 ± 8%	124 ± 4%	107 ± 9%
Bis(2-ethylhexyl) terephthalate	88 ± 1%	86 ± 12%	106 ± 24%	83 ± 10%	106 ± 11%	105 ± 8%	121 ± 3%	106 ± 9%
**Additional compounds**								
Naphthalene	114 ± 17%	137 ± 33%	97 ± 15%	83 ± 29%	87 ± 8%	80 ± 4%	72 ± 9%	70 ± 12%
2-Phenoxyethanol	106 ± 11%	102 ± 5%	99 ± 20%	98 ± 13%	99 ± 9%	103 ± 14%	92 ± 6%	87 ± 8%
Benzothiazole	105 ± 4%	103 ± 6%	106 ± 12%	98 ± 13%	99 ± 7%	101 ± 6%	94 ± 7%	84 ± 17%
Biphenyl	116 ± 7%	108 ± 8%	118 ± 21%	84 ± 10%	96 ± 2%	92 ± 2%	91 ± 6%	70 ± 13%
3-Nitrophenol	96 ± 16%	94 ± 6%	99 ± 24%	94 ± 14%	112 ± 4%	105 ± 5%	97 ± 4%	97 ± 5%
2,4-Dinitrophenol	99 ± 35%	105 ± 18%	100 ± 36%	99 ± 13%	105 ± 6%	93 ± 4%	103 ± 19%	96 ± 7%
4-Nitrophenol	101 ± 16%	104 ± 11%	100 ± 27%	94 ± 13%	111 ± 8%	103 ± 3%	102 ± 9%	88 ± 3%
Diethyl benzyl phosphonate	86 ± 7%	82 ± 13%	106 ± 21%	81 ± 14%	110 ± 21%	109 ± 8%	124 ± 11%	103 ± 12%
Tributyl phosphate	86 ± 3%	84 ± 6%	107 ± 16%	79 ± 14%	102 ± 14%	110 ± 6%	125 ± 7%	102 ± 12%
Benzophenone	97 ± 7%	97 ± 4%	102 ± 16%	94 ± 11%	103 ± 1%	105 ± 1%	102 ± 1%	91 ± 6%

2-Hydroxybenzothiasole	105 ± 15%	102 ± 5%	96 ± 22%	100 ± 17%	110 ± 11%	104 ± 4%	98 ± 4%	87 ± 8%
Benzyl benzoate	99 ± 6%	104 ± 5%	100 ± 14%	95 ± 11%	102 ± 1%	101 ± 3%	95 ± 5%	88 ± 8%
Tris(2-ethylhexyl) phosphate	84 ± 1%	83 ± 11%	103 ± 23%	85 ± 11%	109 ± 18%	101 ± 11%	128 ± 7%	110 ± 15%
Triphenyl phosphate	89 ± 7%	83 ± 9%	104 ± 24%	86 ± 11%	107 ± 13%	105 ± 4%	122 ± 2%	106 ± 7%

Table S5: Repeated thermal desorption of polyester

		Repeated desorption, 175 °C		Continued desorption, 220 °C		
**Name**	**RT (min)**	**1st**	**2nd**	**3rd**	**4th**	**5th**
**Internal standards**						
Quinoline-d_7	5.8	100 ± 4%	n.d	n.d	n.d	n.d
2-Methylbenzothiazole	6.5	100 ± 4%	n.d	n.d	n.d	n.d
3-Nitroaniline-d_4	8.6	100 ± 5%	n.d	n.d	n.d	n.d
Diethyl phthalate-d_4	9.8	100 ± 6%	n.d	n.d	n.d	n.d
4-Nitroaniline-$^{15}N_2$	10.0	100 ± 7%	n.d	n.d	n.d	n.d
Benzophenone-d_{10}	10.3	100 ± 3%	n.d	n.d	n.d	n.d
2,4-Dinitrobromobenzene-d_3	10.6	100 ± 8%	n.d	n.d	n.d	n.d
Eicosane-d_{42}	13.8	100 ± 6%	n.d	n.d	n.d	n.d
Bis (2-ethylhexyl) phthalate-d_4	18.7	100 ± 10%	n.d	n.d	n.d	n.d
**Quinolines**						
Quinoline	5.8	100 ± 9%	n.d	n.d	n.d	n.d
Isoquinoline	6.1	100 ± 10%	n.d	n.d	n.d	n.d
2-Methylquinoline	6.6	100 ± 2%	n.d	n.d	n.d	n.d
8-Methylquinoline	6.7	100 ± 6%	n.d	n.d	n.d	n.d
6-Methylquinoline	7.2	100 ± 8%	n.d	n.d	n.d	n.d
3-Methylquinoline	7.2	100 ± 3%	n.d	n.d	n.d	n.d
4-Methylquinoline	7.4	100 ± 8%	n.d	n.d	n.d	n.d
2,6-Dimethylquinoline	7.9	100 ± 7%	n.d	n.d	n.d	n.d
2,4-Dimethylquinoline	8.2	100 ± 3%	n.d	n.d	n.d	n.d
**Arylamines**						
4-Chloroaniline	5.3	100 ± 6%	n.d	n.d	n.d	n.d
2-Bromoaniline	5.5	100 ± 12%	n.d	n.d	n.d	n.d
3-Bromoaniline	6.4	100 ± 8%	n.d	n.d	n.d	n.d
4-Bromoaniline	6.4	100 ± 7%	n.d	n.d	n.d	n.d
2-Nitroaniline	7.7	100 ± 6%	n.d	n.d	n.d	n.d
3,4-Dichloroaniline	8.0	100 ± 4%	n.d	n.d	n.d	n.d
3-Nitroaniline	8.6	100 ± 11%	n.d	n.d	n.d	n.d
2,6-Dichloro-1,4-phenylenediaminediamine	9.9	100 ± 12%	n.d	n.d	n.d	n.d
4-Chloro-2-nitroaniline	10.0	100 ± 6%	n.d	n.d	n.d	n.d
4-Nitroaniline	10.0	100 ± 6%	n.d	n.d	n.d	n.d
Dibenzylamine	11.4	100 ± 12%	n.d	n.d	n.d	n.d
2-Chloro-4-nitroaniline	11.0	100 ± 6%	n.d	n.d	n.d	n.d
2,6-Dichloro-4-nitroaniline	11.3	100 ± 4%	n.d	n.d	n.d	n.d
2,6-Dibromo-4-nitroaniline	13.5	100 ± 3%	n.d	n.d	n.d	n.d
6-Chloro-2,4-dinitroaniline	13.2	100 ± 6%	n.d	n.d	n.d	n.d
2,4-Dinitroaniline	13.6	100 ± 7%	n.d	n.d	n.d	n.d
2-Bromo-4,6-dinitroaniline	14.2	100 ± 12%	n.d	n.d	n.d	n.d
**Nitrobenzenes**						
3,5-Dichloronitrobenzene	6.8	100 ± 7%	n.d	n.d	n.d	n.d
1,4-Dinitrobenzene	8.0	100 ± 3%	n.d	n.d	n.d	n.d

1,3-Dinitrobenzene	8.2	100 ± 8%	n.d	n.d	n.d	n.d
1,2-Dinitrobenzene	8.4	100 ± 7%	n.d	n.d	n.d	n.d
2,5-Dinitrochlorobenzene	9.3	100 ± 4%	n.d	n.d	n.d	n.d
2,4-Dinitrochlorobenzene	9.5	100 ± 10%	n.d	n.d	n.d	n.d
3,5-Dinitrobromobenzene	10.2	100 ± 5%	n.d	n.d	n.d	n.d
**Phthalates**						
Dimethyl phthalate	8.2	100 ± 3%	n.d	n.d	n.d	n.d
Dimethyl terephthalate	8.8	100 ± 2%	n.d	n.d	n.d	n.d
Dipropyl phthalate	11.8	100 ± 5%	n.d	n.d	n.d	n.d
Isobutyl phthalate	12.7	100 ± 4%	n.d	n.d	n.d	n.d
Benzyl butyl phthalate	17.2	100 ± 2%	n.d	n.d	n.d	n.d
Bis(2-ethylhexyl) phthalate	18.7	100 ± 2%	n.d	n.d	n.d	n.d
Dioctyl phthalate	20.2	100 ± 2%	n.d	n.d	n.d	n.d
Bis(2-ethylhexyl) terephthalate	20.3	100 ± 5%	n.d	n.d	n.d	n.d
**Additional compounds**						
Naphthalene	5.2	99 ± 8%	1 ± 1%	n.d	n.d	n.d
2-Phenoxyethanol	5.5	100 ± 10%	n.d	n.d	n.d	n.d
Benzothiazole	5.7	100 ± 6%	n.d	n.d	n.d	n.d
Biphenyl	7.5	100 ± 5%	n.d	n.d	n.d	n.d
3-Nitrophenol	8.4	100 ± 8%	n.d	n.d	n.d	n.d
2,4-Dinitrophenol	8.9	100 ± 17%	n.d	n.d	n.d	n.d
4-Nitrophenol	9.0	100 ± 5%	n.d	n.d	n.d	n.d
Tributyl phosphate	10.4	100 ± 3%	n.d	n.d	n.d	n.d
Benzophenone	10.4	100 ± 9%	n.d	n.d	n.d	n.d
2-Hydroxybenzothiazole	10.8	100 ± 3%	n.d	n.d	n.d	n.d
Benzyl benzoate	11.9	100 ± 5%	n.d	n.d	n.d	n.d
Triphenyl phosphate	17.7	100 ± 5%	n.d	n.d	n.d	n.d
Tris(2-ethylhexyl) phosphate	18.1	100 ± 3%	n.d	n.d	n.d	n.d

Table S6: Repeated primary desorption from 100 % cotton (Cotton-1)

		Repeated desorption			
**Name**	**RT (min)**	**175 °C**	**220 °C**	**260 °C**	**280 °C**
**Internal standards**					
Quinoline-d_7	5.8	20 ± 3%	24 ± 3%	42 ± 5%	15 ± 2%
2-Methylbenzothiazole	6.5	18 ± 2%	20 ± 4%	38 ± 8%	24 ± 3%
3-Nitroaniline-d_4	8.6	66 ± 22%	19 ± 5%	14 ± 3%	n.d
Diethyl phthalate-d_4	9.8	66 ± 6%	11 ± 3%	15 ± 2%	8 ± 1%
4-Nitroaniline-$^{15}N_2$	10.0	54 ± 14%	28 ± 8%	18 ± 4%	n.d
Benzophenone-d_{10}	10.3	48 ± 9%	15 ± 4%	23 ± 6%	14 ± 2%
2,4-Dinitrobromobenzene-d_3	10.6	84 ± 16%	16 ± 3%	n.d	n.d
Eicosane-d_{42}	13.8	100%	n.d	n.d	n.d
Bis(2-ethylhexyl) phthalate-d_4	18.7	99 ± 2%	n.d	n.d	n.d
**Qunolines**					
Quinoline	5.8	20 ± 4%	20 ± 2%	37 ± 5%	23 ± 2%
Isoquinoline	6.1	26 ± 4%	23 ± 3%	36 ± 5%	16 ± 3%
2-Methylquinoline	6.6	30 ± 8%	25 ± 4%	33 ± 5%	11 ± 1%
8-Methylquinoline	6.7	29 ± 4%	22 ± 3%	31 ± 4%	18 ± 2%
6-Methylquinoline	7.2	29 ± 5%	20 ± 2%	32 ± 4%	20 ± 3%
3-Methylquinoline	7.2	25 ± 4%	21 ± 2%	33 ± 5%	21 ± 3%
4-Methylquinoline	7.4	38 ± 7%	20 ± 3%	28 ± 4%	14 ± 2%
2,6-Dimethylquinoline	7.9	43 ± 7%	21 ± 4%	26 ± 3%	10 ± 1%
2,4-Dimethylquinoline	8.2	56 ± 6%	20 ± 3%	19 ± 3%	5 ± 0.3%
**Arylamines**					
4-Chloroaniline	5.3	78 ± 10%	22 ± 6%	n.d	n.d
2-Bromoaniline	5.5	36 ± 3%	34 ± 7%	27 ± 5%	3 ± 1%
3-Bromoaniline	6.4	78 ± 8%	22 ± 5%	n.d	n.d
4-Bromoaniline	6.4	85 ± 12%	15 ± 4%	n.d	n.d
2-Nitroaniline	7.7	30 ± 7%	27 ± 4%	38 ± 7%	4 ± 1%
3,4-Dichloroaniline	8.0	86 ± 15%	11 ± 3%	3 ± 1%	n.d
3-Nitroaniline	8.6	67 ± 19%	20 ± 5%	13 ± 3%	n.d
2,6-Dichloro-1,4-phenylenediamine	9.9	100 ± 30%	n.d	n.d	n.d
4-Chloro-2-nitroaniline	10.0	55 ± 12%	20 ± 5%	24 ± 6%	2 ± 0.3%
4-Nitroaniline	10.0	52 ± 15%	24 ± 8%	24 ± 10%	n.d
Dibenzylamine	11.4	98 ± 13%	2 ± 1%	n.d	n.d
2-Chloro-4-nitroaniline	11.0	51 ± 11%	21 ± 4%	25 ± 5%	3 ± 1%
2,6-Dichloro-4-nitroaniline	11.3	65 ± 15%	14 ± 5%	19 ± 6%	2 ± 1%
2,6-Dibromo-4-nitroaniline	13.5	78 ± 14%	11 ± 3%	11 ± 5%	n.d
6-Chloro-2,4-dinitroaniline	13.2	74 ± 11%	14 ± 4%	12 ± 7%	n.d
2,4-Dinitroaniline	13.6	51 ± 17%	18 ± 6%	31 ± 12%	n.d
2-Bromo-4,6-dinitroaniline	14.2	71 ± 9%	19 ± 5%	9 ± 2%	n.d
**Nitrobenzenes**					
3,5-Dichloronitrobenzene	6.8	57 ± 7%	27 ± 5%	17 ± 4%	n.d
1,4-Dinitrobenzene	8.0	51 ± 11%	43 ± 10%	6 ± 3%	n.d
1,3-Dinitrobenzene	8.2	47 ± 12%	36 ± 8%	17 ± 4%	n.d

1,2-Dinitrobenzene	8.4	59 ± 10%	31 ± 8%	10 ± 2%	n.d
2,5-Dinitrochlorobenzene	9.2	79 ± 17%	21 ± 10%	n.d	n.d
2,4-Dinitrochlorobenzene	9.5	83 ± 24%	17 ± 8%	n.d	n.d
3,5-Dinitrobromobenzene	10.2	80 ± 13%	17 ± 5%	3 ± 1%	n.d
**Phthalates**					
Dimethyl phthalate	8.2	48 ± 7%	19 ± 3%	25 ± 5%	8 ± 1%
Dimethyl terephthalate	8.8	33 ± 7%	24 ± 3%	33 ± 4%	10 ± 1%
Dipropyl phthalate	11.8	87 ± 7%	5 ± 1%	5 ± 1%	3 ± 1%
Isobutyl phthalate	12.7	94 ± 3%	2 ± 1%	2 ± 1%	n.d
Benzyl butyl phthalate	17.2	90 ± 7%	5 ± 1%	4 ± 1%	n.d
Bis(2-ethylhexyl) phthalate	18.7	98 ± 4%	n.d	n.d	n.d
Dioctyl phthalate	20.2	99 ± 5%	n.d	n.d	n.d
Bis(2-ethylhexyl) terephthalate	20.3	99 ± 3%	n.d	n.d	n.d
**Additional compounds**					
Naphthalene	5.2	19 ± 4%	23 ± 1%	39 ± 6%	19 ± 4%
2-Phenoxyethanol	5.5	12 ± 2%	16 ± 4%	41 ± 7%	31 ± 4%
Benzothiazole	5.7	14 ± 2%	21 ± 3%	41 ± 5%	24 ± 4%
Biphenyl	7.5	38 ± 5%	22 ± 4%	26 ± 3%	14 ± 2%
3-Nitrophenol	8.4	39 ± 12%	26 ± 6%	31 ± 6%	3 ± 1%
2,4-Dinitrophenol	8.9	64 ± 23%	21 ± 6%	15 ± 8%	n.d
4-Nitrophenol	9.0	31 ± 9%	24 ± 6%	38 ± 7%	7 ± 1%
Tributyl phosphate	10.4	97 ± 13%	2 ± 1%	1 ± 1%	n.d
Benzophenone	10.4	55 ± 8%	14 ± 5%	20 ± 6%	11 ± 3%
2-Hydroxybenzothiazole	10.8	28 ± 7%	18 ± 5%	35 ± 7%	20 ± 3%
Benzyl benzoate	11.9	70 ± 11%	10 ± 3%	14 ± 5%	6 ± 1%
Triphenyl phosphate	17.7	92 ± 3%	5 ± 1%	2 ± 1%	n.d
Tris(2-ethylhexyl) phosphate	18.1	99 ± 3%	n.d	n.d	n.d

Table S7: Repeated desorption from Cotton-1 at 175 °C followed by 220 °C

	Desorption at 175 °C			Continued desorption at 220 °C		
**Name**	**1st**	**2nd**	**3rd**	**4th**	**5th**	**6th**
			**Internal standards**			
**Quinoline-d_7**	36 ± 5%	1.4 ± 0.5%	n.d	10 ± 2%	1.5 ± 0.2%	n.d
**2-Methylbensothiasole**	44 ± 6%	1.4 ± 0.4%	n.d	8 ± 2%	1.2 ± 0.1%	n.d
**3-Nitroaniline-d_4**	29 ± 3%	n.d	n.d	n.d	n.d	n.d
**Diethyl phthalate-d_4**	81 ± 5%	n.d	n.d	1.7 ± 0.4%	n.d	n.d
**4-Nitroaniline-$^{15}N_2$**	33 ± 4%	n.d	n.d	n.d	n.d	n.d
**Benzophenone-d_{10}**	75 ± 4%	n.d	n.d	3.1 ± 0.3%	n.d	n.d
**2,4-Dinitrobromobenzene-d_3**	57 ± 5%	n.d	n.d	n.d	n.d	n.d
**Eicosane-d_{42}**	100 %	n.d	n.d	n.d	n.d	n.d
**Bis(2-ethylhexyl) phthalate-d_4**	134 ± 11%	1.3 ± 0.5%	n.d	n.d	n.d	n.d
			**Quinolines**			
**Quinoline**	35 ± 5%	1.3 ± 0.4%	n.d	11 ± 2.3%	1.5 ± 0.1%	n.d
**Isoquinoline**	36 ± 4%	1.1 ± 0.4%	n.d	7 ± 1.6%	n.d	n.d
**2-Methylquinoline**	47 ± 4%	1.4 ± 0.4%	n.d	8 ± 1.7%	n.d	n.d
**8-Methylquinoline**	55 ± 8%	1.3 ± 0.4%	n.d	6 ± 1.2%	n.d	n.d
**6-Methylquinoline**	49 ± 3%	1.2 ± 0.4%	n.d	8 ± 1.7%	n.d	n.d
**3-Methylquinoline**	46 ± 3%	1.2 ± 0.4%	n.d	8 ± 1.7%	n.d	n.d
**4-Methylquinoline**	52 ± 3%	1.1 ± 0.3%	n.d	6.4 ± 1.38%	n.d	n.d
**2,6-Dimethylquinoline**	57 ± 4%	1.1 ± 0.3%	n.d	5.7 ± 1.1%	n.d	n.d
**2,4-Dimethylquinoline**	62 ± 4%	n.d	n.d	4.3 ± 0.78%	n.d	n.d
			**Arylamines**			
**4-Chloroaniline**	26 ± 6%	n.d	n.d	n.d	n.d	n.d
**2-Bromoaniline**	37 ± 7%	1.1 ± 0.4%	n.d	1.6 ± 0.46%	n.d	n.d
**3-Bromoaniline**	34 ± 6%	n.d	n.d	n.d	n.d	n.d
**4-Bromoaniline**	27 ± 5%	n.d	n.d	n.d	n.d	n.d
**2-Nitroaniline**	42 ± 2%	1.1 ± 0.5%	n.d	6 ± 1.2%	n.d	n.d
**3,4-Dichloroaniline**	45 ± 5%	n.d	n.d	n.d	n.d	n.d
**3-Nitroaniline**	29 ± 4%	n.d	n.d	n.d	n.d	n.d
**2,6-Dichloro-1,4-phenylenediamine**	4 ± 1%	n.d	n.d	n.d	n.d	n.d
**4-Chloro-2-nitroaniline**	59 ± 5%	n.d	n.d	2.1 ± 0.3%	n.d	n.d
**4-Nitroaniline**	33 ± 4%	n.d	n.d	1.1 ± 0.2%	n.d	n.d
**Dibenzylamine**	55 ± 5%	n.d	n.d	n.d	n.d	n.d
**2-Chloro-4-nitroaniline**	56 ± 4%	n.d	n.d	2.3 ± 0.3%	n.d	n.d
**2,6-Dichloro-4-nitroaniline**	71 ± 5%	n.d	n.d	1.8 ± 0.3%	n.d	n.d
**6-Chloro-2,4-dinitroaniline**	79 ± 7%	n.d	n.d	1.2 ± 0.3%	n.d	n.d
**2,6-Dibromo-4-nitroaniline**	77 ± 5%	n.d	n.d	2 ± 0.4%	n.d	n.d
**2,4-Dinitroaniline**	68 ± 6%	n.d	n.d	1.9 ± 0.6%	n.d	n.d
**2-Bromo-4,6-dinitroaniline**	96 ± 9%	n.d	n.d	2.3 ± 0.5%	n.d	n.d
			**Nitrobenzens**			
**3,5-Dichloronitrobenzene**	54 ± 9%	n.d	n.d	1.96 ± 0.5%	n.d	n.d
**1,4-Dinitrobenzene**	34 ± 4%	n.d	n.d	3.3 ± 0.9%	n.d	n.d
**1,3-Dinitrobenzene**	43 ± 5%	n.d	n.d	3.75 ± 0.8%	n.d	n.d
**1,2-Dinitrobenzene**	52 ± 4%	n.d	n.d	2.52 ± 0.5%	n.d	n.d
**2,5-Dinitrochlorobenzene**	53 ± 4%	n.d	n.d	n.d	n.d	n.d

**2,4-Dinitrochlorobenzene**	55 ± 4%	n.d	n.d	n.d	n.d	n.d
**3,5-Dinitrobromobenzene**	60 ± 6%	n.d	n.d	n.d	n.d	n.d
**Phthalates**						
**Dimethyl phthalate**	64 ± 5%	n.d	n.d	3.95 ± 0.5%	n.d	n.d
**Dimethyl terephthalate**	43 ± 4%	1.1 ± 0.4%	n.d	6 ± 1.3%	n.d	n.d
**Dipropyl phthalate**	87 ± 6%	n.d	n.d	n.d	n.d	n.d
**Isobutyl phthalate**	90 ± 7%	n.d	n.d	n.d	n.d	n.d
**Benzyl butyl phthalate**	137 ± 12%	n.d	n.d	n.d	n.d	n.d
**Bis(2-ethylhexyl) phthalate**	100 ± 19%	4.2 ± 1.2%	5 ± 2%	6.81 ± 1.5%	2 ± 0%	n.d
**Dioctyl phthalate**	101 ± 9%	5.2 ± 1.4%	2 ± 1%	n.d	n.d	n.d
**Bis(2-ethylhexyl) terephthalate**	89 ± 10%	5.5 ± 1.4%	2 ± 1%	n.d	n.d	n.d
**Additional compounds**						
**Naphthalene**	30 ± 11%	1.9 ± 0.4%	1 ± 0.2%	3.7 ± 0.7%	n.d	n.d
**2-Phenoxyethanol**	29 ± 1%	1.4 ± 0.6%	n.d	12 ± 1.5%	1.9 ± 0.2%	n.d
**Bensothiasole**	33 ± 6%	1.3 ± 0.5%	n.d	9.2 ± 2%	1.7 ± 0.2%	n.d
**Biphenyl**	63 ± 9%	1.3 ± 0.3%	n.d	2.8 ± 0.6%	n.d	n.d
**3-Nitrophenol**	50 ± 4%	n.d	n.d	5 ± 1.4%	n.d	n.d
**2,4-Dinitrophenol**	59 ± 6%	n.d	n.d	2.5 ± 0.9%	n.d	n.d
**4-Nitrophenol**	48 ± 3%	n.d	n.d	4.7 ± 1.6%	n.d	n.d
**Tributyl phosphate**	93 ± 8%	n.d	n.d	n.d	n.d	n.d
**Benzophenone**	75 ± 5%	n.d	n.d	2.7 ± 0.1%	n.d	n.d
**2-Hydroxybenzothiazole**	53 ± 3%	n.d	n.d	4.7 ± 1.5%	n.d	n.d
**Benzyl benzoate**	82 ± 6%	n.d	n.d	1.6 ± 0.1%	n.d	n.d
**Tris(2-ethylhexyl) phosphate**	127 ± 10%	n.d	n.d	n.d	n.d	n.d
**Triphenyl phosphate**	151 ± 8%	2.6 ± 1.2%	n.d	n.d	n.d	n.d

Table S8: Quantification of native compounds in Textile 1 – 4 with ATD at 175 °C

Values are given as mean conc. (RSD %) in µg/g. Relative Response Method uses Cotton-1 as external standard matrix

Name	SE-GC/MS	ATD-GC/MS "IS Response Method"	ATD-GC/MS "Relative Response Method"	MDL
**Textile 1: 95% Cotton, 5% Elastane**				
2-Phenoxyethanol	0.2 (17)	det.	0.3 (14)	0.08
Benzothiazole	0.1 (31)	det.	det.	0.11
Quinoline	0.5 (48)	0.6 (4)	0.5 (11)	0.03
Isoquinoline	0.2 (53)	det.	det.	0.05
2-Methylquinoline	0.1 (49)	det.	det.	0.04
8-Methylquinoline	0.02 (56)	n.d.	n.d.	0.03
6-Methylquinoline	0.1 (55)	det.	det.	0.04
Biphenyl	0.05 (49)	n.d.	n.d.	0.01
3-Methylquinoline	0.03 (54)	det.	n.d.	0.02
4-Methylquinoline	0.03 (56)	det.	det.	0.02
2,6-Dimethylquinoline	0.04 (56)	n.d.	n.d.	0.01
2,4-Dimethylquinoline	0.04 (54)	n.d.	n.d.	0.01
Dimethyl phthalate	0.03 (9)	n.d.	n.d.	0.02
Dimethyl terephthalate	0.1 (35)	det.	n.d.	0.05
Tributyl phosphate	0.04 (5)	det.	n.d.	0.03
Benzophenone	0.1 (11)	det.	det.	0.04
4-Chloro-2-nitroaniline	0.05 (51)	n.d.	n.d.	0.13
2-Chloro-4-nitroaniline	0.6 (30)	det.	det.	0.34
Benzyl benzoate	0.4 (9)	0.4 (5)	0.3 (6)	0.04
2,6-Dichloro-4-nitroaniline	0.04 (89)	n.d.	n.d.	0.26
Isobutyl phthalate	2 (8)	1.3 (4)	1.9 (6)	0.01
Benzyl butyl phthalate	0.2 (11)	det.	n.d.	0.27
Bis(2-ethylhexyl) phthalate	0.8 (7)	0.8 (5)	0.8 (6)	0.004
**Textile 2: 100% Cotton**				
2-Phenoxyethanol	0.3 (33)	det.	0.3 (1)	0.08
Benzothiazole	0.1 (43)	n.d.	n.d.	0.11
Quinoline	0.7 (89)	0.7 (11)	0.6 (4)	0.03
Isoquinoline	0.1 (104)	det.	det.	0.05
2-Methylquinoline	0.1 (94)	det.	n.d.	0.04
8-Methylquinoline	0.02 (97)	det.	n.d.	0.03
6-Methylquinoline	0.1 (97)	n.d.	n.d.	0.04
Biphenyl	0.03 (40)	det.	det.	0.01
3-Methylquinoline	0.02 (97)	n.d.	n.d.	0.02
4-Methylquinoline	0.02 (84)	n.d.	n.d.	0.02
2,6-Dimethylquinoline	0.02 (93)	n.d.	det.	0.01
Dimethyl phthalate	0.01 (8)	n.d.	n.d.	0.02
Dimethyl terephthalate	0.003 (10)	n.d.	n.d.	0.05
Tributyl phosphate	0.01 (18)	n.d.	n.d.	0.03

Benzophenone	0.02 (75)	n.d.	n.d.	0.04
4-Chloro-2-nitroaniline	0.2 (0)	det.	det.	0.13
Benzyl benzoate	0.3 (12)	0.2 (4)	0.2 (2)	0.04
Isobutyl phthalate	0.3 (79)	0.3 (5)	0.2 (3)	0.01
Benzyl butyl phthalate	0.2 (9)	n.d.	n.d.	0.27
Bis(2-ethylhexyl) phthalate	0.6 (29)	1.5 (3)	0.5 (2)	0.004
Triphenyl phosphate	0.2 (11)	n.d.	det.	0.03
Bis(2-ethylhexyl) terephthalate	0.2 (13)	n.d.	n.d.	1.45
**Textile 3: 100% Cotton**				
2-Phenoxyethanol	0.2 (14)	0.3 (11)	det.	0.08
Benzothiazole	0.04 (17)	n.d.	n.d.	0.11
Quinoline	0.1 (28)	det.	det.	0.03
Isoquinoline	0.02 (31)	n.d.	n.d.	0.05
2-Methylquinoline	0.02 (29)	n.d.	n.d.	0.04
8-Methylquinoline	0 (46)	n.d.	n.d.	0.03
6-Methylquinoline	0.01 (43)	n.d.	n.d.	0.04
Biphenyl	0.01 (32)	det.	det.	0.01
4-Methylquinoline	0.01 (48)	n.d.	n.d.	0.02
Dimethyl phthalate	0.02 (4)	n.d.	n.d.	0.02
Dimethyl terephthalate	0.01 (5)	n.d.	n.d.	0.05
Tributyl phosphate	0.1 (10)	det.	det.	0.03
Benzophenone	0.1 (16)	n.d.	n.d.	0.04
Benzyl benzoate	0.1 (7)	0.1 (11)	det.	0.04
Isobutyl phthalate	1 (26)	1.2 (3)	0.8 (21)	0.01
Benzyl butyl phthalate	0.1 (21)	n.d.	n.d.	0.27
Bis(2-ethylhexyl) phthalate	0.6 (3)	0.5 (2)	0.5 (19)	0.004
**Textile 4: 100% Cotton**				
4-Chloroaniline	0.12 (7)	n.d.	det.	0.09
2-Phenoxyethanol	1.2 (16)	0.86 (11)	1.2 (5)	0.08
Benzothiazole	0.14 (17)	det.	det.	0.11
Quinoline	0.06 (18)	det.	det.	0.03
Dimethyl phthalate	0.03 (12)	det.	det.	0.02
Dimethyl terephthalate	0.02 (14)	n.d.	n.d.	0.05
Tributyl phosphate	0.02 (7)	n.d.	n.d.	0.03
Benzophenone	0.14 (10)	det.	det.	0.04
Benzyl benzoate	0.36 (16)	0.33 (3)	0.26 (4)	0.04
2,6-Dichloro-4-nitroaniline	0.14 (20)	n.d.	n.d.	0.26
Isobutyl phthalate	4.8 (7)	5.3 (2)	3.8 (9)	0.01
6-Chloro-2,4-dinitroaniline	6.7 (13)	4.0 (1)	6.1 (3)	0.18
Bis(2-ethylhexyl) phthalate	1.1 (48)	1.1 (5)	0.77 (7)	0.004
Triphenyl phosphate	0.15 (46)	0.23 (2)	0.24 (10)	0.03
Bis(2-ethylhexyl) terephthalate	0.37 (9)	n.d.	n.d.	1.45

Table S9: Linearity within calibration range 1-100 µg/g with ATD at 175 °C

	Polyester			Cotton-1			Cotton-2		
**Compounds**	**RT (min)**	**R^2**	**RMSE (%)**	**R^2**	**RMSE (%)**	**Ratio to polyester**	**R^2**	**RMSE (%)**	**Ratio to polyester**
**Quinolines**									
Quinoline	5.8	0.988	15%	0.982	12%	0.36	0.944	30%	0.27
Isoquinoline	6.1	0.982	21%	0.973	18%	0.44	0.964	28%	0.32
2-Methylquinoline	6.6	0.989	8%	0.984	13%	0.63	0.958	23%	0.50
8-Methylquinoline	6.7	0.990	20%	0.984	15%	0.52	0.983	12%	0.46
6-Methylquinoline	7.1	0.993	8%	0.987	15%	0.66	0.959	24%	0.51
3-Methylquinoline	7.2	0.991	8%	0.988	11%	0.65	0.964	21%	0.48
4-Methylquinoline	7.4	0.983	17%	0.993	11%	0.70	0.984	15%	0.51
2,6-Dimethylquinoline	7.9	0.989	9%	0.994	15%	0.80	0.985	18%	0.62
2,4-Dimethylquinoline	8.2	0.979	14%	0.991	15%	0.73	0.979	16%	0.56
**Arylamines**									
4-Chloroaniline	5.3	0.983	22%	0.996	11%	0.27	0.939	28%	0.23
2-Bromoaniline	5.5	0.995	31%	0.995	10%	0.25	0.960	16%	0.22
3-Bromoaniline	6.4	0.991	18%	0.978	19%	0.47	0.954	27%	0.35
4-Bromoaniline	6.4	0.987	16%	0.980	18%	0.43	0.937	35%	0.33
2-Nitroaniline	7.7	0.975	38%	0.986	18%	0.62	0.961	38%	0.42
3,4-Dichloroaniline	8.0	0.989	9%	0.996	22%	0.69	0.981	26%	0.60
3-Nitroaniline	8.6	0.975	37%	0.982	36%	0.62	0.971	71%	0.39
2,6-Dichloro-1,4-phenylenediamine	9.9	0.986	27%	0.957	42%	0.66	0.942	79%	0.43
4-Chloro-2-nitroaniline	10.0	0.983	31%	0.989	17%	0.75	0.968	30%	0.56
4-Nitroaniline	10.0	0.978	25%	0.992	29%	0.61	0.960	43%	0.40
Dibenzylamine	11.4	0.980	38%	0.991	25%	0.68	0.990	13%	0.53
2-Chloro-4-nitroaniline	11.0	0.978	43%	0.989	18%	0.74	0.966	32%	0.51
2,6-Dichloro-4-nitroaniline	11.3	0.991	22%	0.992	13%	0.79	0.979	21%	0.62
3,5-Dibromo 1,2-phenylenediamine	12.7	0.947	29%	0.932	34%	0.48	0.928	46%	0.25

6-Chloro-2,4-dinitroaniline	13.5	0.951	108%	0.978	18%	0.73	0.957	39%	0.49
2,6-Dibromo-4-nitroaniline	13.6	0.975	48%	0.992	9%	0.75	0.978	18%	0.59
2,4-Dinitroaniline	14.2	0.922	104%	0.983	22%	0.76	0.959	63%	0.43
2-Bromo-4,6-dinitroaniline	15.4	0.934	111%	0.988	13%	0.84	0.971	26%	0.56
**Nitrobenzenes**									
3,5-Dichloronitrobenzene	6.8	0.991	7%	0.973	25%	0.40	0.963	18%	0.28
1,4-Dinitrobenzene	8.0	0.981	44%	0.961	35%	0.58	0.923	77%	0.36
1,3-Dinitrobenzene	8.2	0.974	35%	0.966	22%	0.70	0.937	60%	0.45
1,2-Dinitrobenzene	8.3	0.967	45%	0.984	17%	0.67	0.970	36%	0.47
2,5-Dinitrochlorobenzene	9.2	0.976	30%	0.992	24%	0.74	0.950	55%	0.57
2,4-Dinitrochlorobenzene	9.5	0.992	14%	0.995	27%	0.69	0.963	49%	0.59
3,5-Dinitrobromobenzene	10.1	0.955	78%	0.979	17%	0.70	0.970	37%	0.52
**Phthalates**									
Dimethyl phthalate	8.2	0.979	21%	0.991	10%	0.76	0.979	16%	0.61
Dimethyl terephthalate	8.8	0.982	10%	0.987	14%	0.67	0.987	28%	0.47
Dipropyl phthalate	11.8	0.989	15%	0.996	13%	0.79	0.990	11%	0.74
Isobutyl phthalate	13.2	0.989	13%	0.998	7%	0.78	0.990	8%	0.80
Benzyl butyl phthalate	17.7	0.974	44%	0.999	4%	0.85	0.994	9%	0.73
Bis(2-ethylhexyl) phthalate	20.2	0.977	37%	0.998	7%	0.78	0.846	19%	0.78
Dioctyl phthalate	20.3	0.988	13%	0.996	10%	0.72	0.993	5%	0.86
Bis(2-ethylhexyl) terephthalate	21.3	0.995	8%	0.996	6%	0.66	0.993	10%	0.82
**Additional compounds**									
1,2-Dihydroxybenzene	5.0	0.954	104%	0.968	29%	0.54	0.945	39%	0.32
Naphthalene	5.2	0.998	7%	0.959	73%	0.15	0.993	25%	0.06
2-Phenoxyethanol	5.4	0.996	38%	0.981	17%	0.49	0.927	39%	0.33
Benzothiazole	5.6	0.992	11%	0.990	14%	0.22	0.945	26%	0.22
Biphenyl	7.4	0.987	17%	0.991	11%	0.45	0.991	21%	0.36
3-Nitrophenol	8.4	0.967	43%	0.979	15%	0.68	0.967	28%	0.43
2,4-Dinitrophenol	8.9	0.966	57%	0.984	16%	0.63	0.957	73%	0.37

4-Nitrophenol	9.0	0.973	35%	0.988	17%	0.65	0.969	36%	0.44
Tributyl phosphate	10.4	0.989	12%	0.995	19%	0.78	0.993	13%	0.80
Diethylbenzylphosphonate	9.6	0.972	67%	0.987	12%	0.76	0.980	16%	0.60
Benzophenone	10.4	0.992	15%	0.996	14%	0.80	0.978	19%	0.68
2-Hydroxybenzothiazole	10.7	0.996	12%	0.995	20%	0.69	0.967	35%	0.46
Benzyl benzoate	11.9	0.990	7%	0.998	15%	0.79	0.990	14%	0.70
Tris(2-ethylhexyl) phosphate	18.1	0.972	46%	0.996	5%	0.86	0.987	7%	0.72
Triphenyl phosphate	18.7	0.998	20%	0.986	16%	0.86	0.990	18%	0.82

Table S10: Instrumental and method detection limits with ATD at 175 °C

Compounds	RT (min)	Polyester LOD/LOQ [ng]	Polyester MDL/MQL [ng/g]	Cotton-1 LOD/LOQ [ng]	Cotton-1 MDL/MQL [ng/g]
**Quinolines**					
Quinoline-d_7	5.7	0.1 / 0.4	7 / 20	0.6 / 1.9	30 / 90
Quinoline	5.8	0.3 / 0.9	10 / 40	0.5 / 1.6	30 / 80
Isoquinoline	6.1	0.4 / 1.1	20 / 50	1 / 3.1	50 / 160
2-Methylquinoline	6.6	0.1 / 0.4	7 / 20	0.8 / 2.3	40 / 120
8-Methylquinoline	6.7	0.2 / 0.5	10 / 20	0.6 / 1.7	30 / 90
6-Methylquinoline	7.1	0.2 / 0.6	10 / 30	0.8 / 2.3	40 / 110
3-Methylquinoline	7.2	0.2 / 0.6	10 / 30	0.5 / 1.4	20 / 70
4-Methylquinoline	7.4	0.1 / 0.4	7 / 20	0.4 / 1.2	20 / 60
2,6-Dimethylquinoline	7.9	0.1 / 0.3	5 / 10	0.2 / 0.7	10 / 30
2,4-Dimethylquinoline	8.2	0.1 / 0.3	5 / 20	0.2 / 0.5	10 / 30
**Arylamines**					
3-Nitroaniline-d_4	8.6	2 / 6	100 / 300	10 / 40	700 / 2100
4-Nitroaniline-$^{15}N_2$	10.0	2 / 6	100 / 300	20 / 60	1000 / 3100
4-Chloroaniline	5.3	0.4 / 1	20 / 100	2 / 6	100 / 300
2-Bromoaniline	5.5	0.1 / 0.4	10 / 20	0.4 / 1	20 / 100
3-Bromoaniline	6.4	0.3 / 1	20 / 50	2 / 6	100 / 300
4-Bromoaniline	6.4	0.7 / 2	40 / 100	2 / 7	100 / 400
2-Nitroaniline	7.7	1.3 / 4	60 / 200	2 / 6	100 / 300
3,4-Dichloroaniline	8.0	1 / 3	50 / 200	4 / 10	200 / 500
3-Nitroaniline	8.6	3.4 / 10	170 / 500	14 / 40	700 / 2200
2,6-Dichloro-1,4-phenylenediamine	9.9	4.7 / 14	230 / 700	60 / 180	2900 / 8900
4-Chloro-2-nitroaniline	10.0	1.5 / 5	80 / 200	3 / 8	100 / 400
4-Nitroaniline	10.0	4.7 / 10	230 / 700	20 / 60	1000 / 2900
Dibenzylamine	11.4	5.7 / 20	280 / 900	20 / 60	1000 / 3100
2-Chloro-4-nitroaniline	11.0	1.1 / 3	60 / 200	7 / 20	300 / 1000
2,6-Dichloro-4-nitroaniline	11.3	2.9 / 9	140 / 400	5 / 16	300 / 800
6-Chloro-2,4-dinitroaniline	13.2	1.5 / 5	70 / 200	4 / 11	200 / 500
2,6-Dibromo-4-nitroaniline	13.5	1.2 / 4	60 / 200	4 / 11	200 / 500
2,4-Dinitroaniline	13.6	8.1 / 20	400 / 1200	30 / 100	1600 / 4900
2-Bromo-4,6-dinitroaniline	14.2	7.2 / 20	400 / 1100	10 / 20	400 / 1100
**Nitrobenzenes**					
2,4-Dinitrobromobenzene-d_3	10.6	1.5 / 5	80 / 230	6.1 / 19	300 / 900
3,5-Dichloronitrobenzene	6.8	0.7 / 2	30 / 100	1.4 / 4	100 / 200
1,4-Dinitrobenzene	8.0	1.7 / 5	80 / 250	3.3 / 10	200 / 500
1,3-Dinitrobenzene	8.2	0.5 / 1	20 / 70	1.4 / 4	100 / 200
1,2-Dinitrobenzene	8.3	1 / 3	50 / 160	3.5 / 11	200 / 500
2,5-Dinitrochlorobenzene	9.2	1 / 3	50 / 140	2.7 / 8	100 / 400
2,4-Dinitrochlorobenzene	9.5	2.8 / 9	140 / 430	4.3 / 13	200 / 700
3,5-Dinitrobromobenzene	10.1	2 / 6	100 / 310	3.8 / 12	200 / 600
**Phthalates**					

Diethyl phthalate-d_4	9.8	0.2 / 0.6	10 / 30	0.5 / 1.4	20 / 100
Bis(2-ethylhexyl) phthalate-d_4	18.7	0.2 / 0.6	10 / 30	1.1 / 3.3	50 / 200
Dimethyl phthalate	8.2	0.2 / 0.5	10 / 30	0.4 / 1.2	20 / 100
Dimethyl terephthalate	8.8	0.5 / 1.6	30 / 80	1 / 3	50 / 100
Dipropyl phthalate	11.8	0.1 / 0.3	10 / 20	0.2 / 0.6	10 / 30
Isobutyl phthalate	12.7	0.1 / 0.2	3 / 10	0.1 / 0.4	10 / 20
Benzyl butyl phthalate	17.2	0.9 / 2.8	50 / 140	5 / 20	270 / 800
Bis(2-ethylhexyl) phthalate	18.7	0.4 / 1.3	20 / 60	0.1 / 0.3	4 / 10
Dioctyl phthalate	20.2	1.2 / 3.7	60 / 200	10 / 30	600 / 2000
Bis(2-ethylhexyl) terephthalate	20.3	2.1 / 6.2	100 / 300	30 / 90	1400 / 4400
**Additional compounds**					
2-Methylbenzothiazole	6.4	0.1 / 0.3	5 / 10	1.1 / 3	60 / 170
Benzophenone-d_{10}	10.3	0.2 / 0.5	9 / 30	0.4 / 1	20 / 60
Naphthalene	5.2	0.1 / 0.4	7 / 20	0.8 / 2	40 / 100
2-Phenoxyethanol	5.4	1 / 3	50 / 150	1.6 / 5	80 / 200
Benzothiazole	5.6	0.4 / 1.1	19 / 60	2.2 / 7	100 / 300
Biphenyl	7.4	0.1 / 0.2	3 / 10	0.2 / 1	10 / 30
3-Nitrophenol	8.4	4.4 / 13	220 / 700	7.5 / 20	370 / 1100
2,4-Dinitrophenol	8.9	2.7 / 8	140 / 400	8.8 / 30	440 / 1300
4-Nitrophenol	9.0	5.9 / 18	300 / 900	11.5 / 30	570 / 1700
Tributyl phosphate	10.4	0.2 / 0.5	10 / 30	0.6 / 1.8	30 / 100
Benzophenone	10.4	0.7 / 2.2	35 / 100	0.8 / 3	40 / 100
2-Hydroxybenzothiazole	10.7	2.6 / 7.9	130 / 400	6.5 / 20	320 / 1000
Benzyl benzoate	11.9	0.4 / 1.3	20 / 70	0.8 / 2.4	40 / 100
Tris(2-ethylhexyl) phosphate	17.7	0.3 / 0.9	10 / 40	0.6 / 1.9	30 / 100
Triphenyl phosphate	18.1	0.4 / 1.1	20 / 50	3.9 / 11.7	190 / 600

Figure S1: Quinolines: Desorbed yield from Cotton-1 and Cotton-2 with ATD at 175 °C

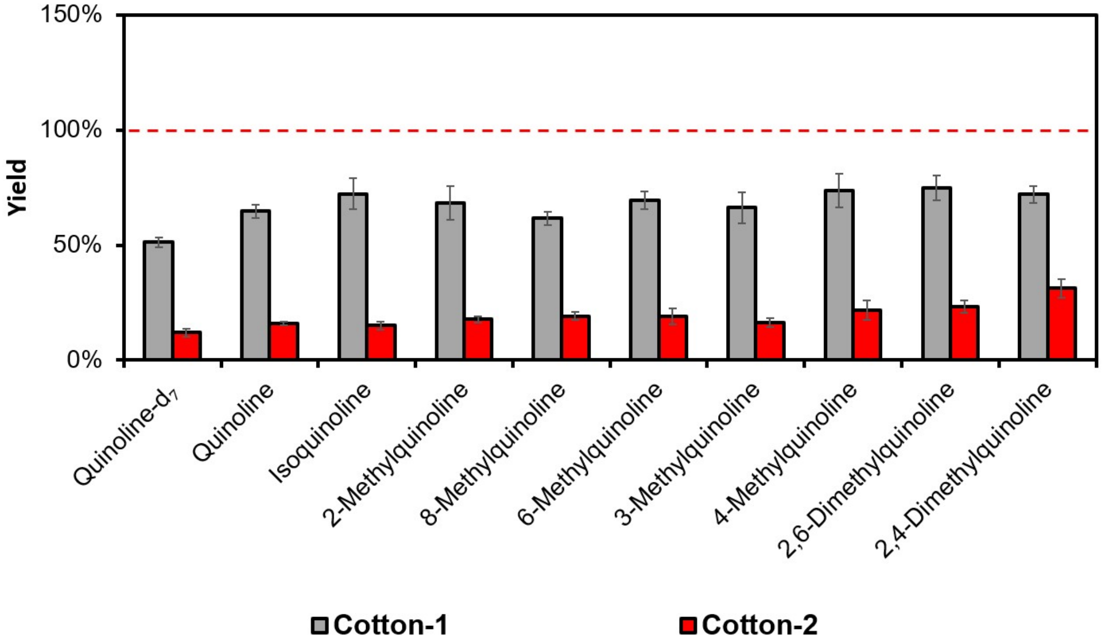

Figure S2: Arylamines: Desorbed yield from Cotton-1 and Cotton-2 with ATD at 175 °C

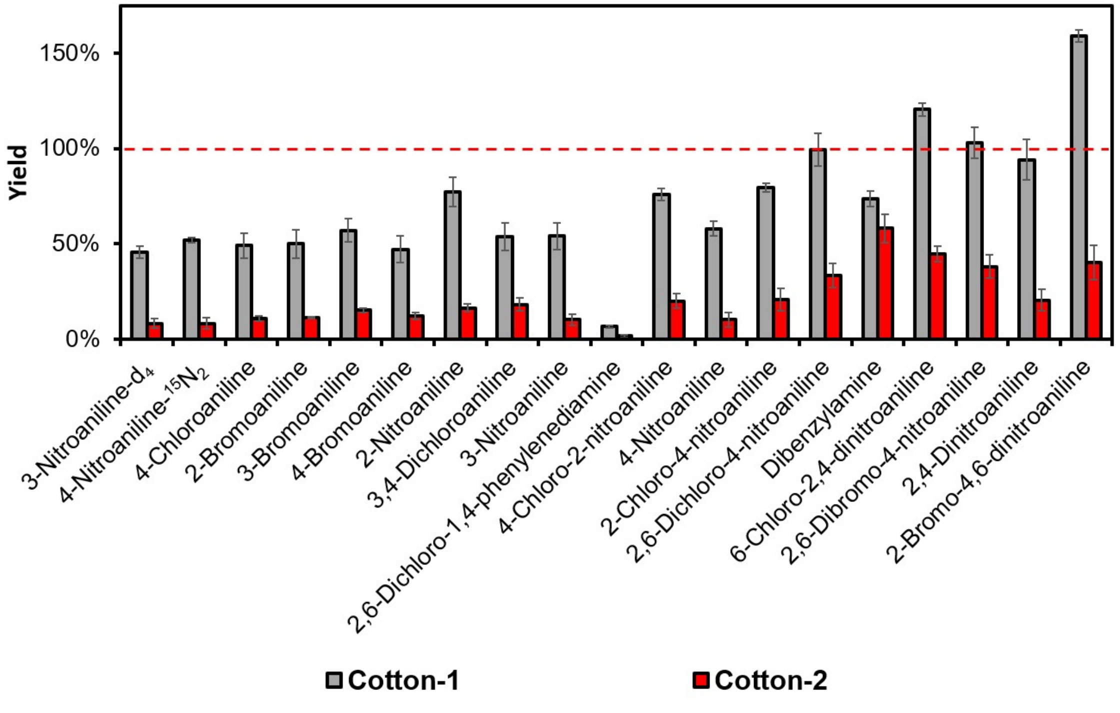

Figure S3: Nitrobenzenes: Desorbed yield from Cotton-1 and Cotton-2 with ATD at 175 °C

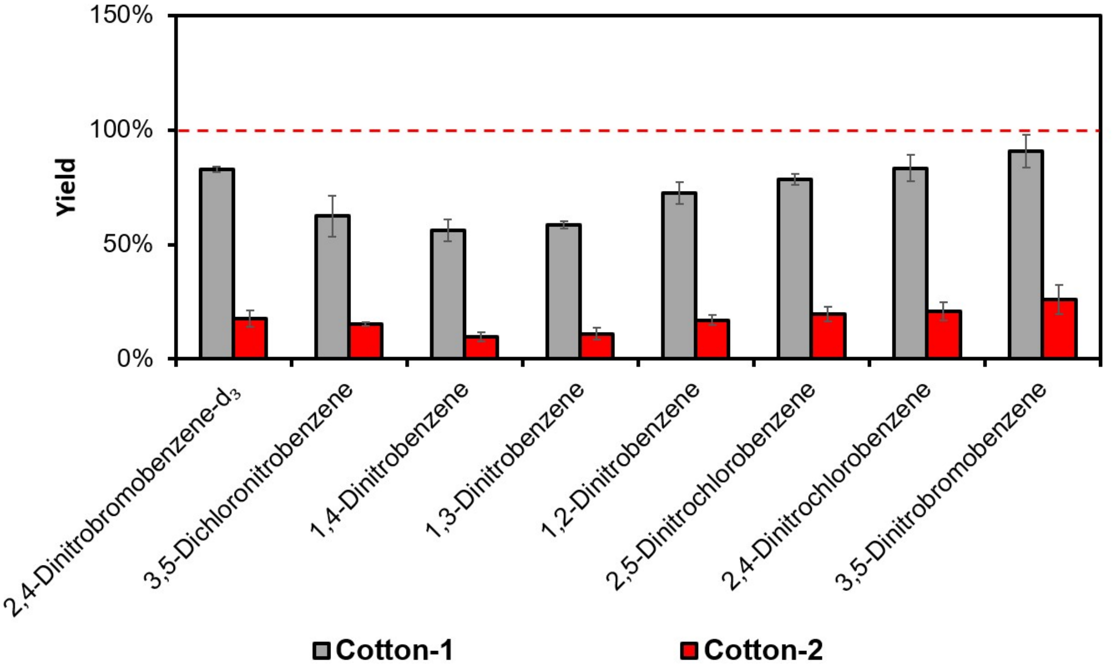

Figure S4: Phthalates: Desorbed yield from Cotton-1 and Cotton-2 with ATD at 175 °C

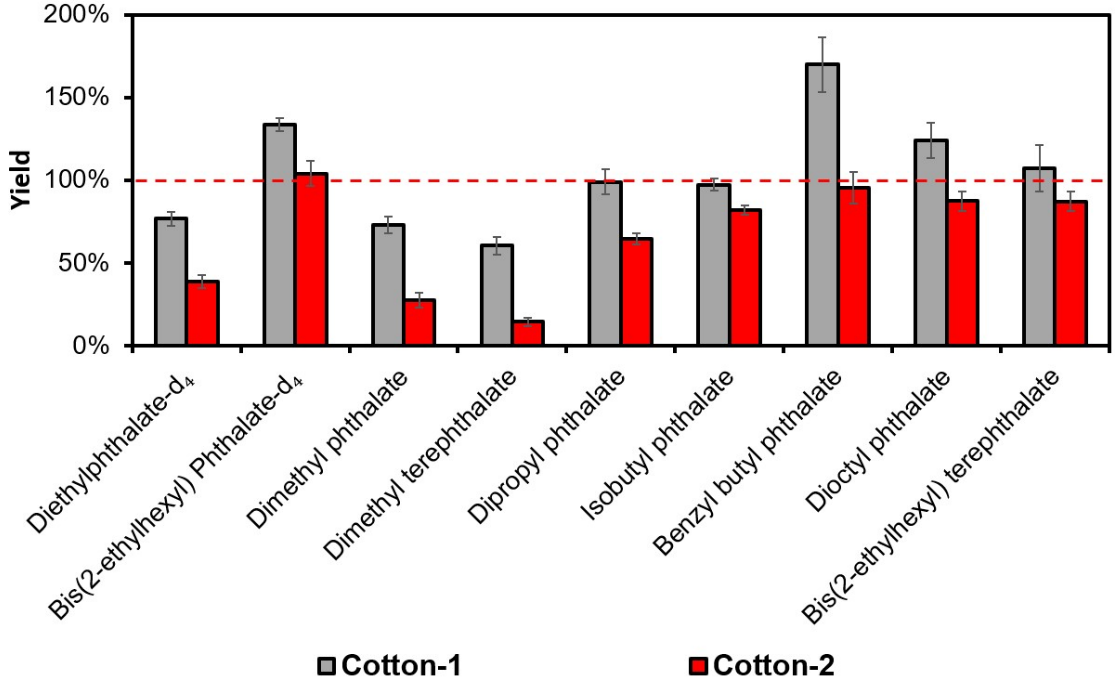

Figure S5: Nitrophenols: Desorbed yield from Cotton-1 and Cotton-2 with ATD at 175 °C

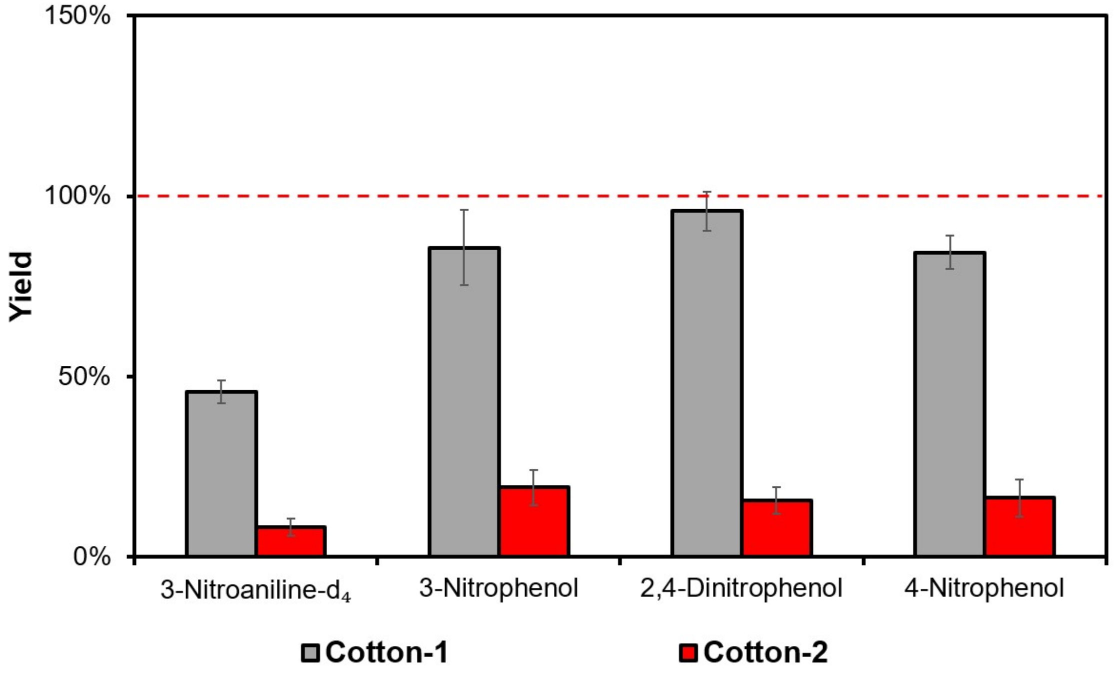

Figure S6: Additional compounds: Desorbed yield from Cotton-1 and Cotton-2 with ATD at 175 °C

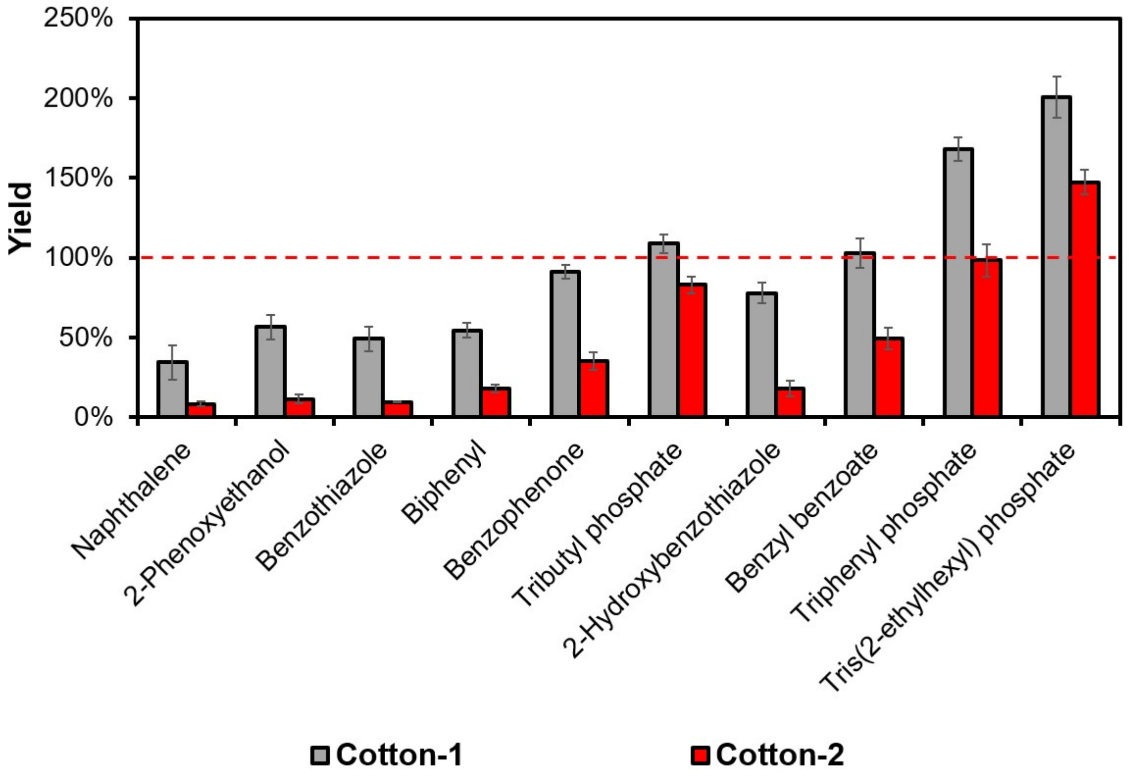

Figure S7: Quinolines: Desorption efficiency from S1-S8 with ATD at 175 °C

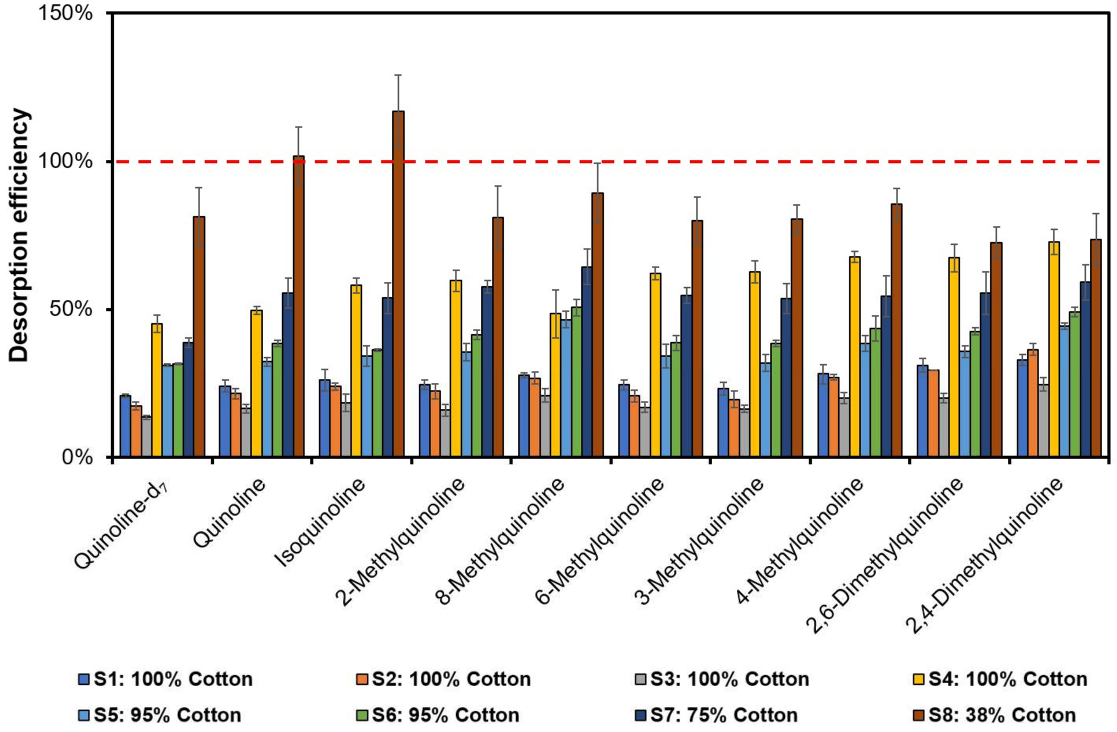

Figure S8: Arylamines: Desorption efficiency from S1-S8 with ATD at 175 °C

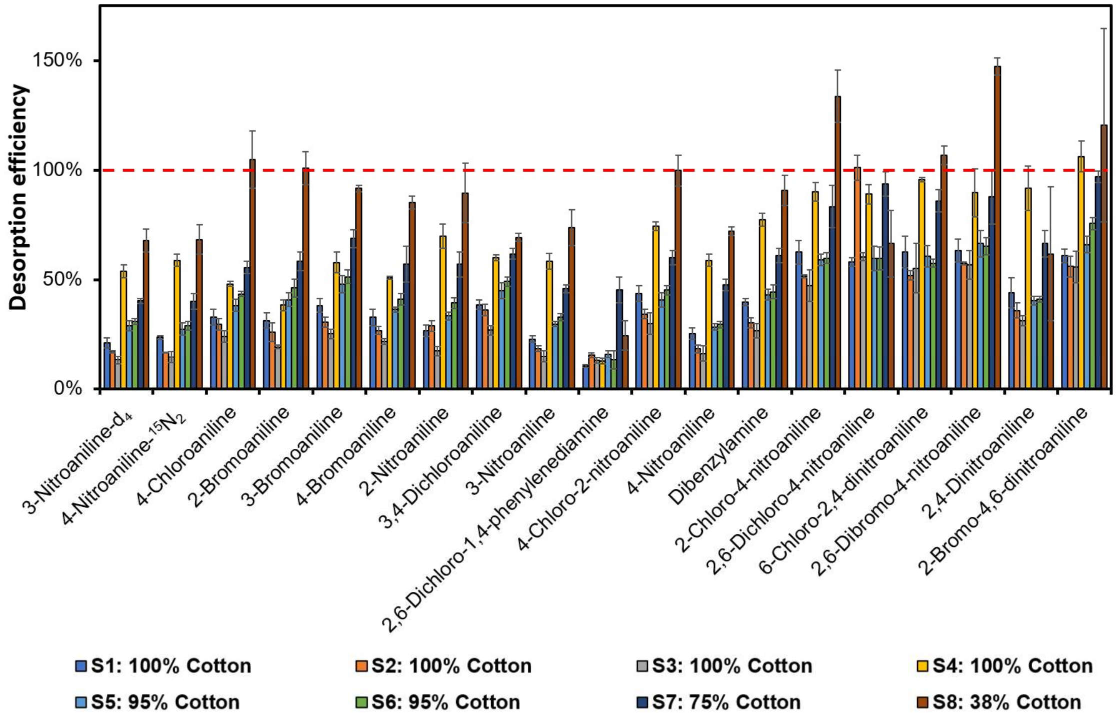

Figure S9: Nitrobenzenes: Desorption efficiency from S1-S8 with ATD at 175 °C

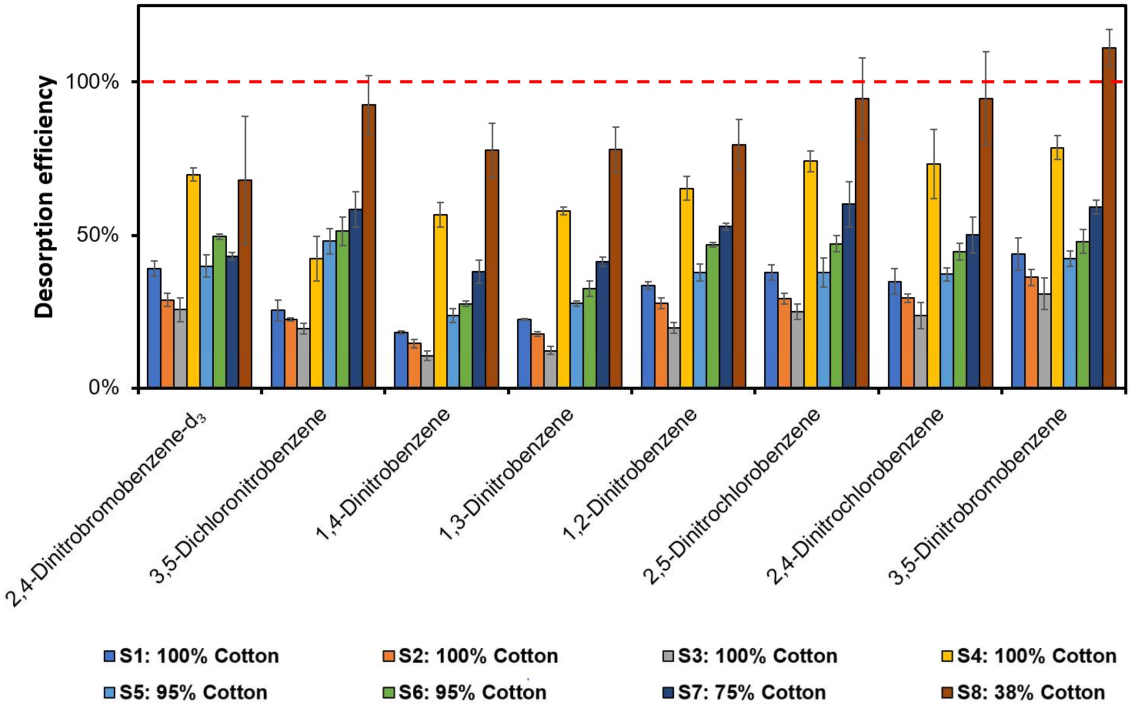

Figure S10: Nitrophenols: Desorption efficiency from S1-S8 with ATD at 175 °C

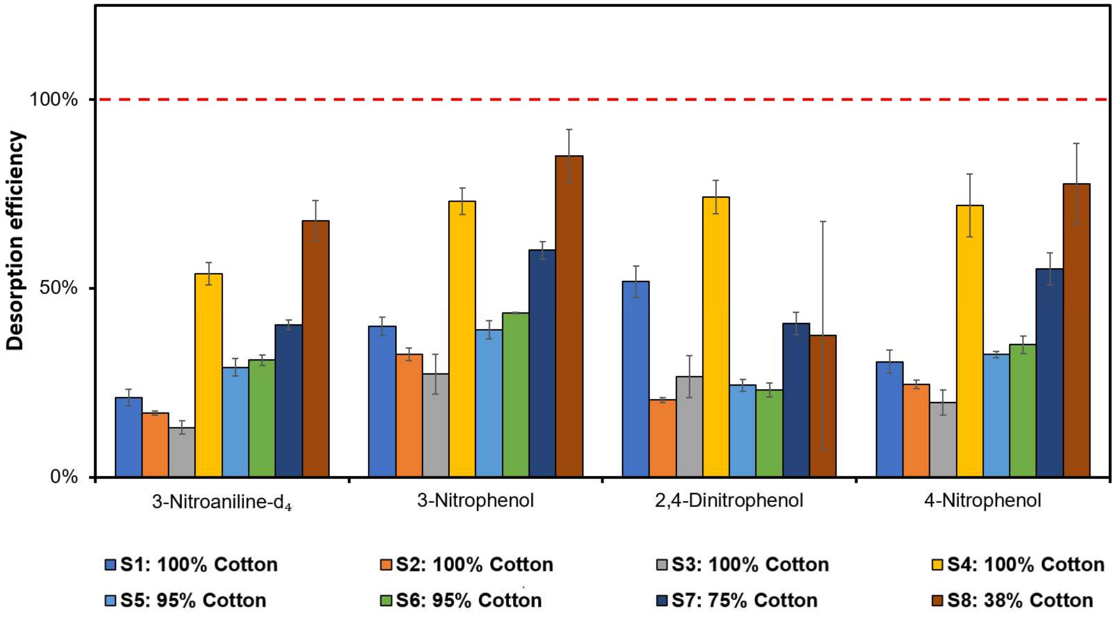

Figure S11: Phthalates: Desorption efficiency from S1-S8 with ATD at 175 °C

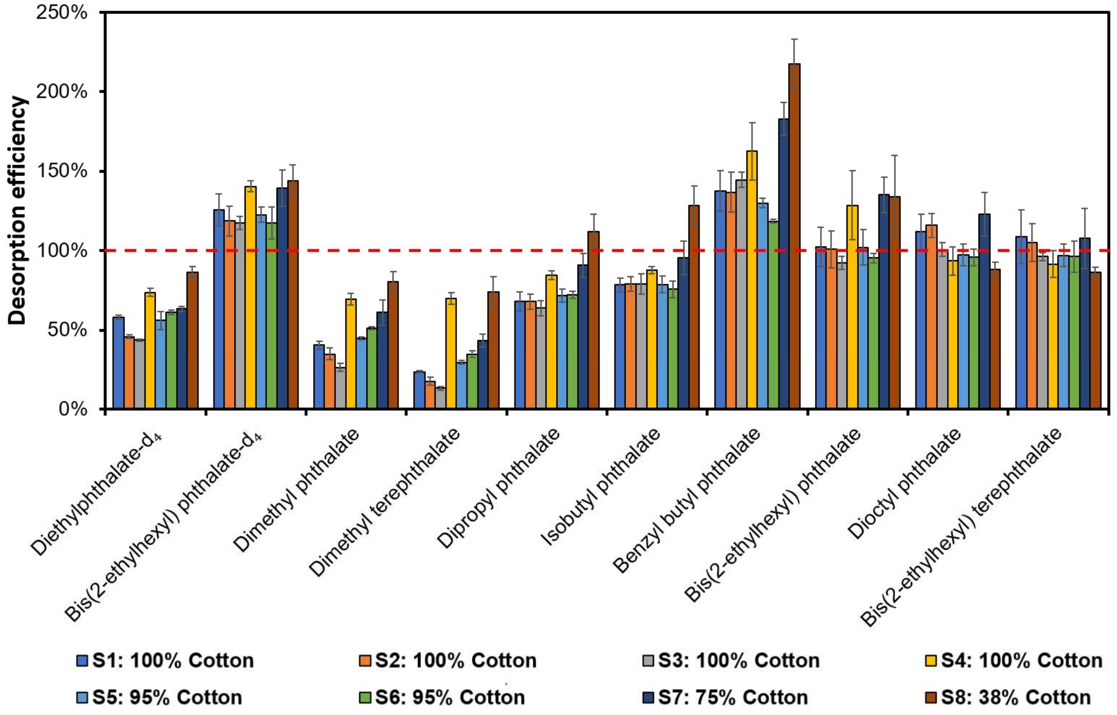

Figure S12: Additional compounds: Desorption efficiency from S1-S8 with ATD at 175 °C

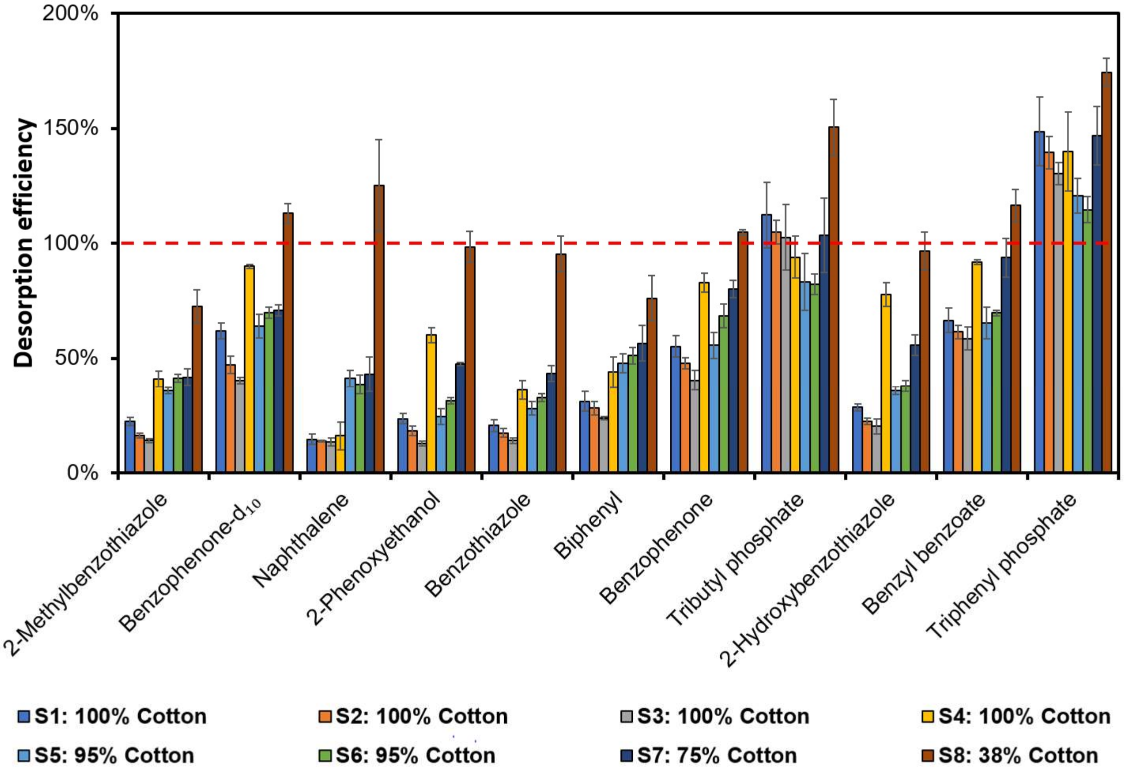

Figure S13: Optical and electronic microscope images of samples S1 -S8. Scale bar: 500 µm

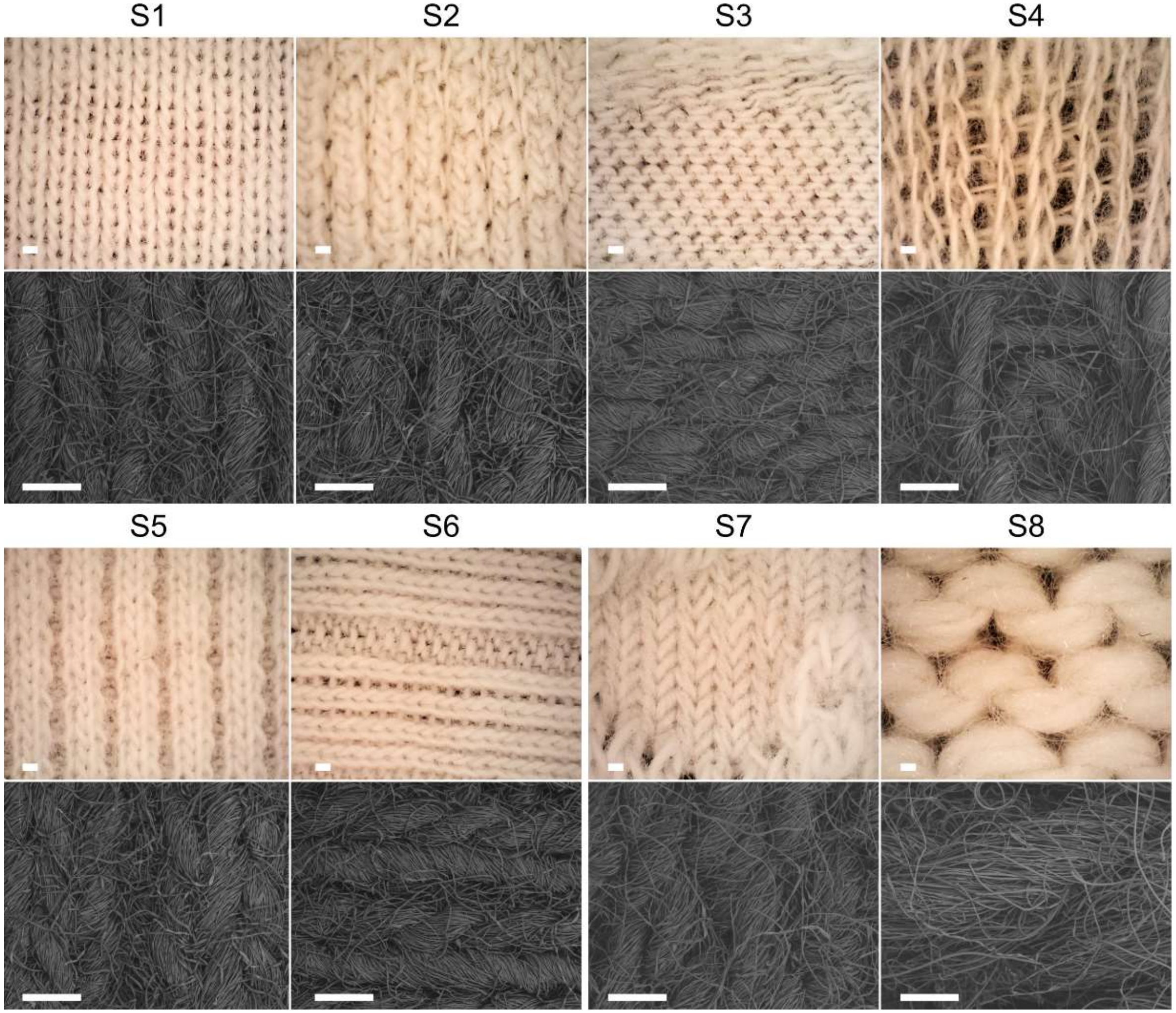

Figure S14: Quinolines: Method accuracy using "Relative Response Method"

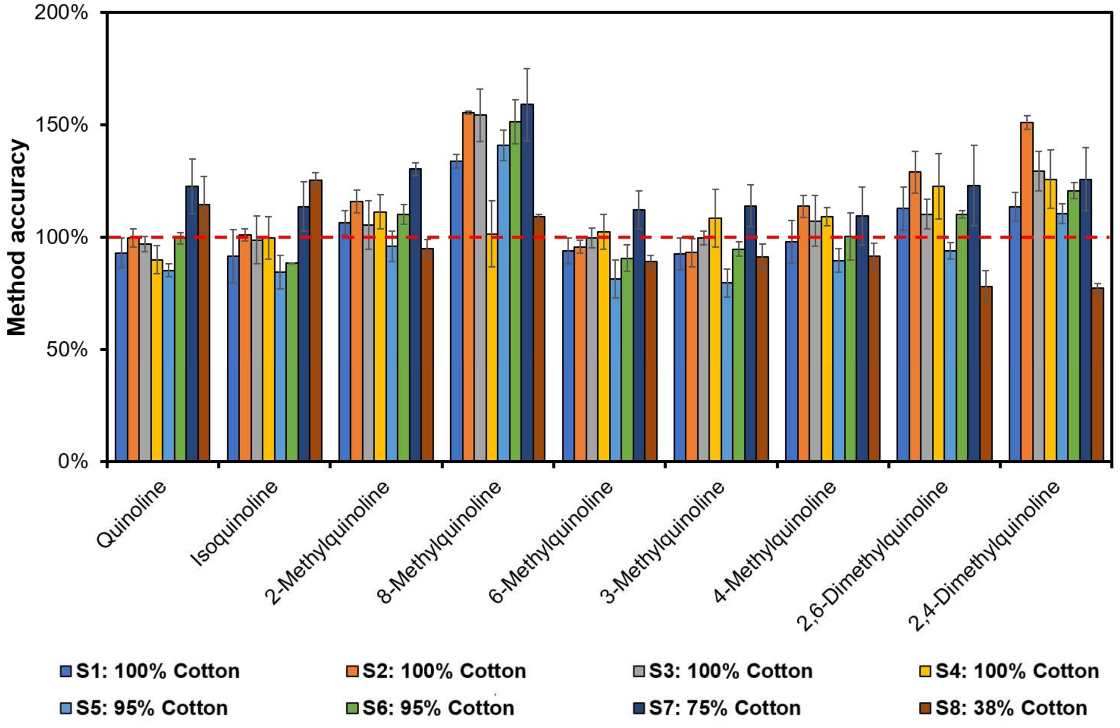

Figure S15: Arylamines - Method accuracy using "Relative Response Method"

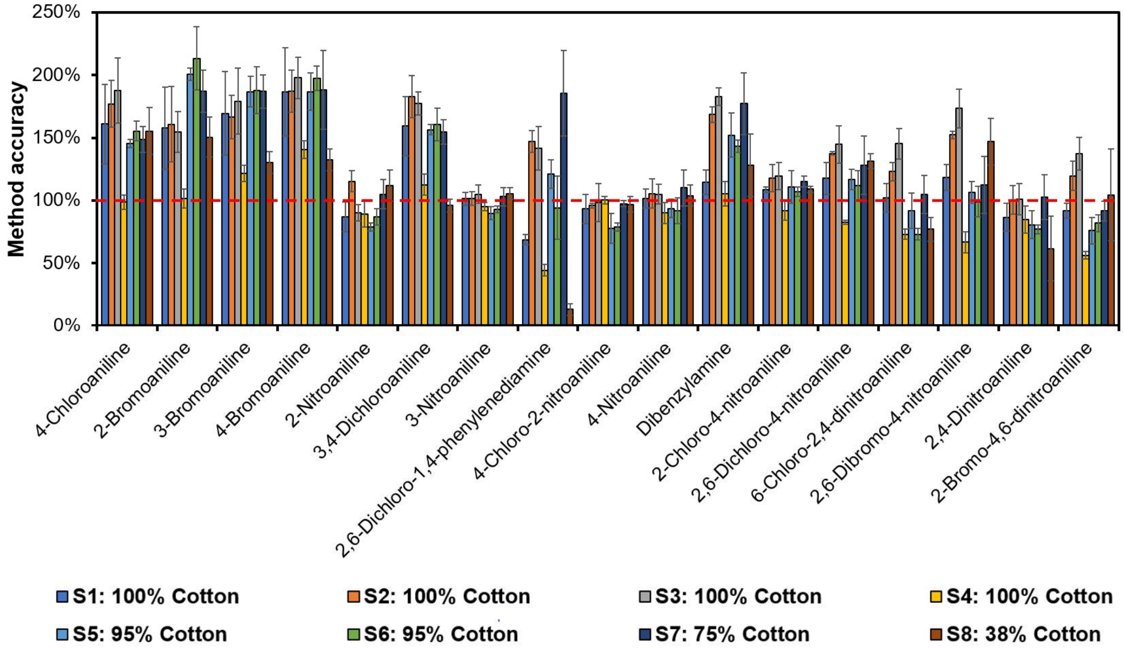

Figure S16: Nitrobenzenes: Method accuracy using "Relative Response Method"

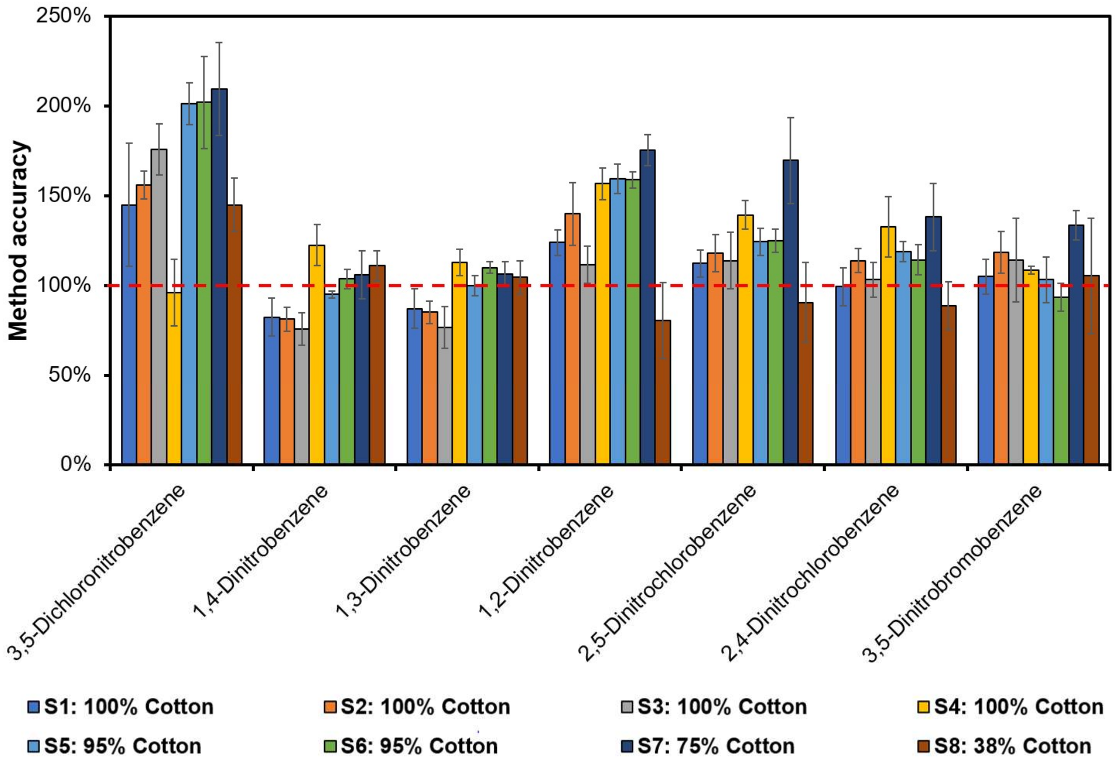

Figure S17: Nitrophenols: Method accuracy using "Relative Response Method"

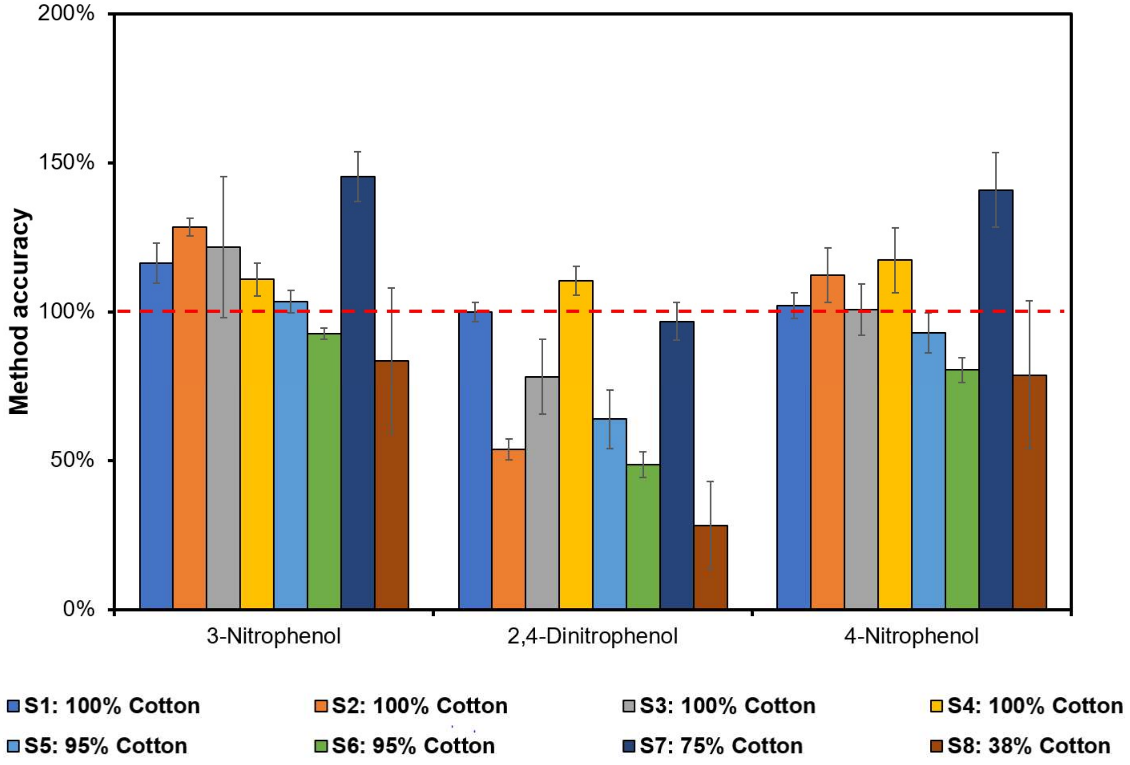

Figure S18: Phthalates: Method accuracy using "Relative Response Method"

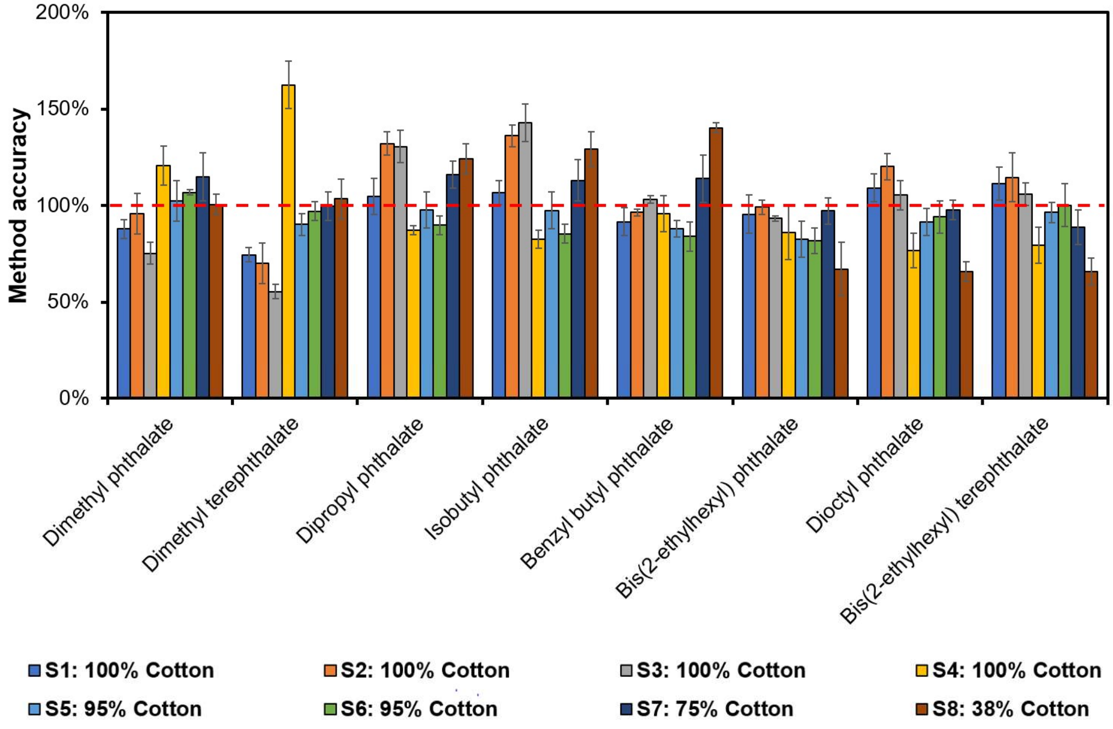

Figure S19: Additional compounds: Method accuracy using "Relative Response Method"

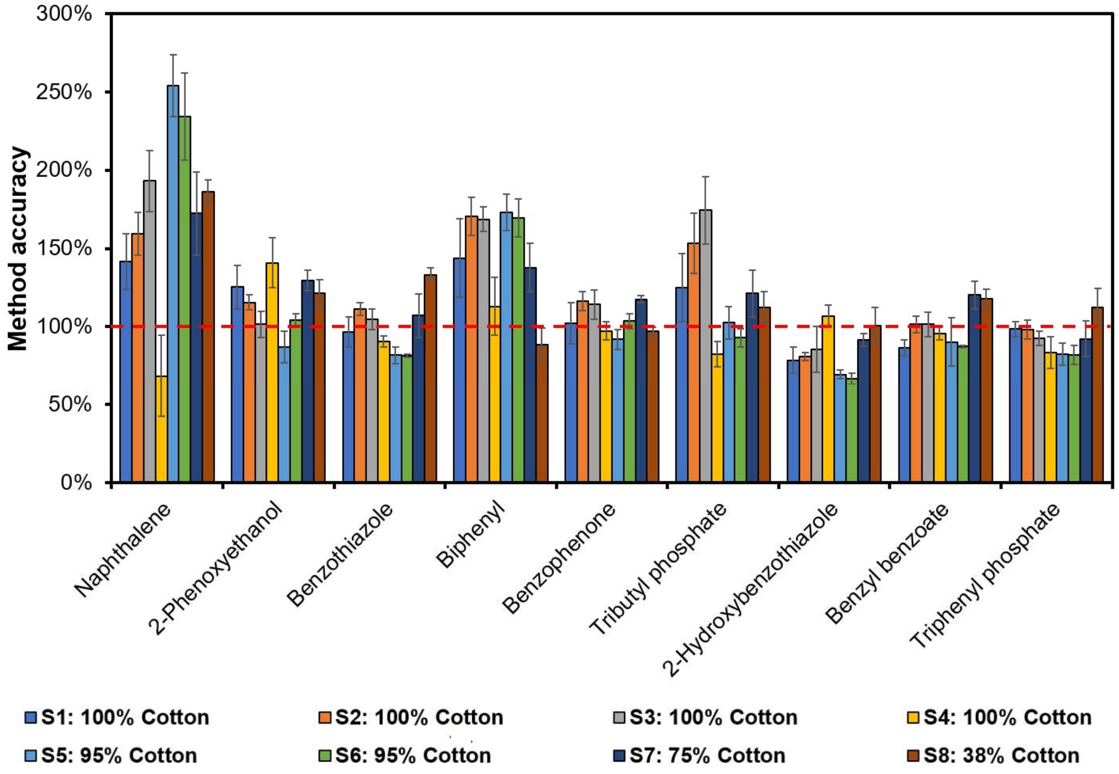

Figure S20: Quinolines: Method accuracy “IS Response Method”

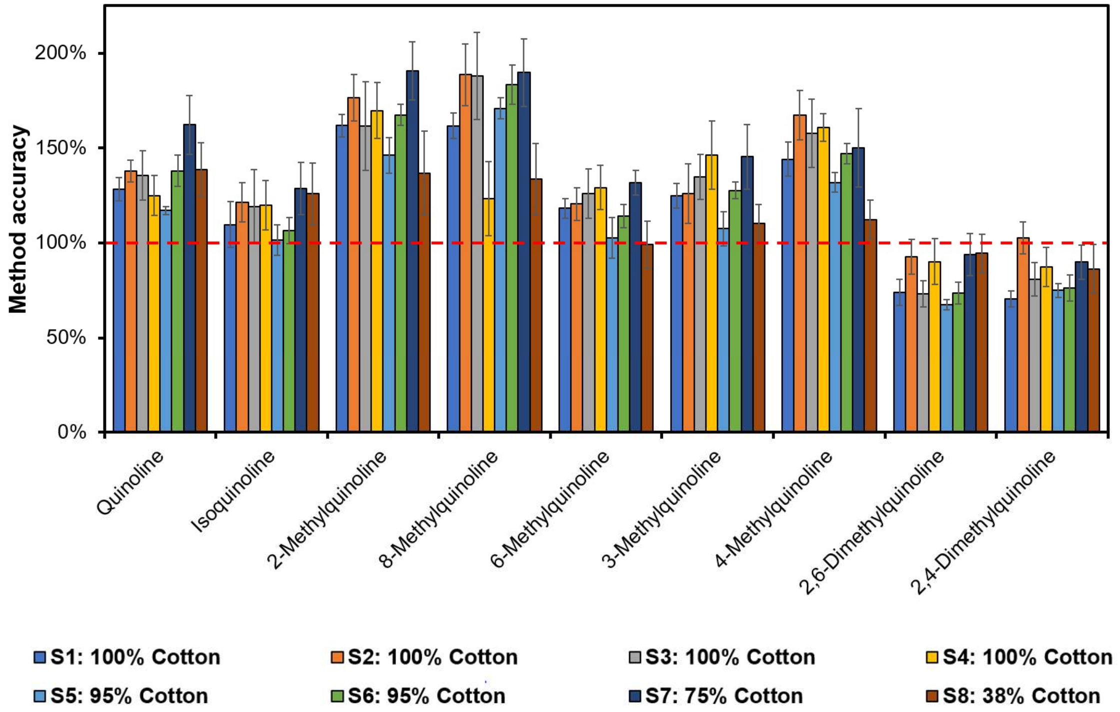

Figure S21: Arylamines: Method accuracy “IS Response Method”

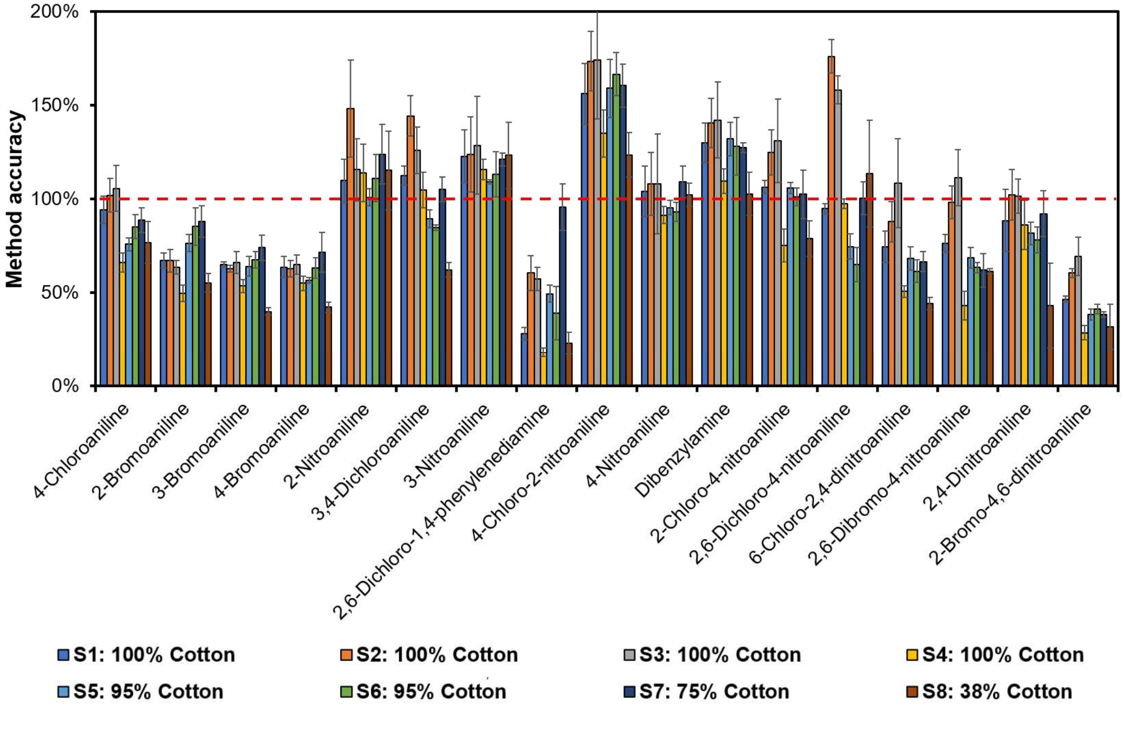

Figure SI22: Nitrobenzenes: Method accuracy “IS Response Method”

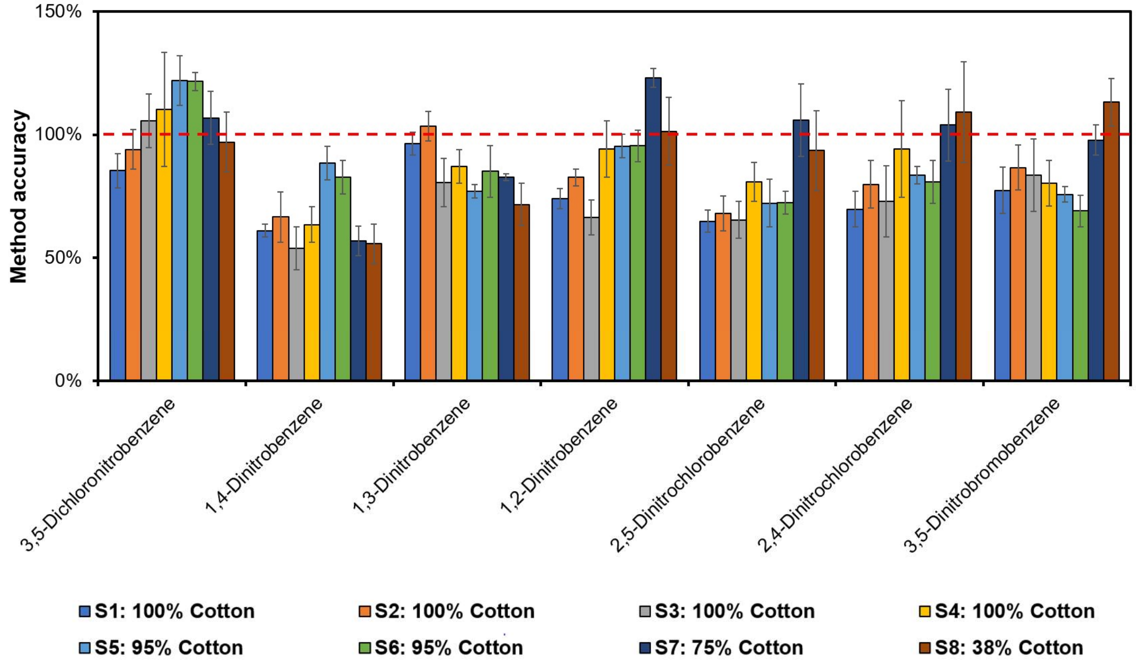

Figure S23: Nitrophenols: Method accuracy “IS Response Method”

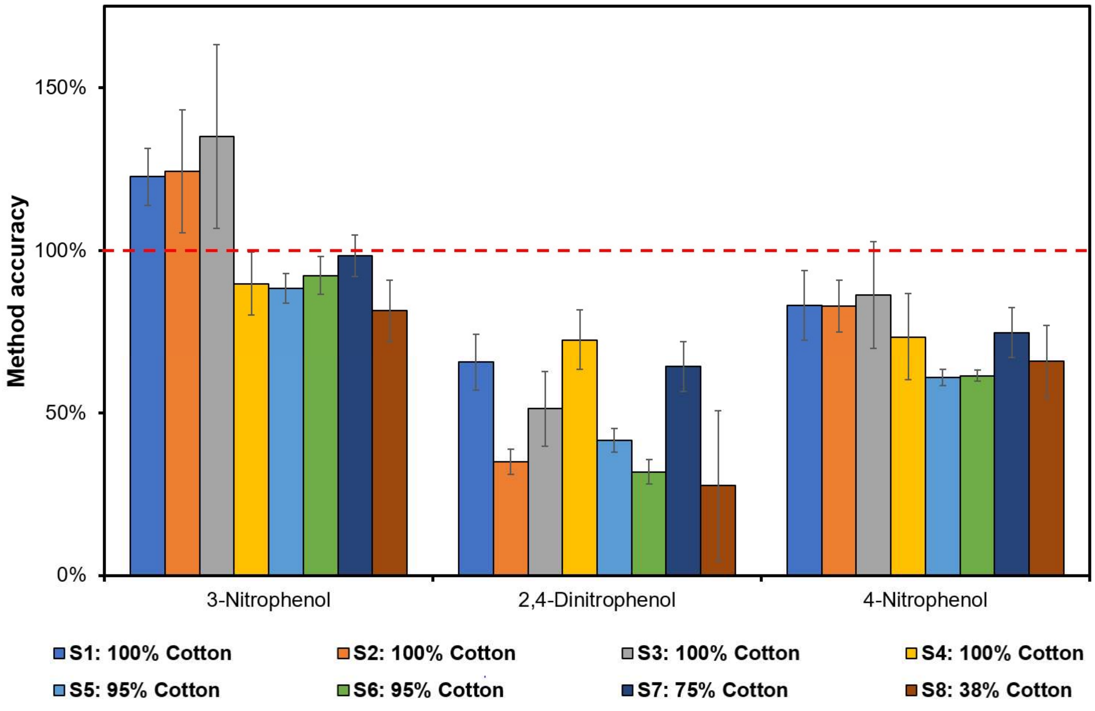

Figure S24: Phthalates: Method accuracy “IS Response Method”

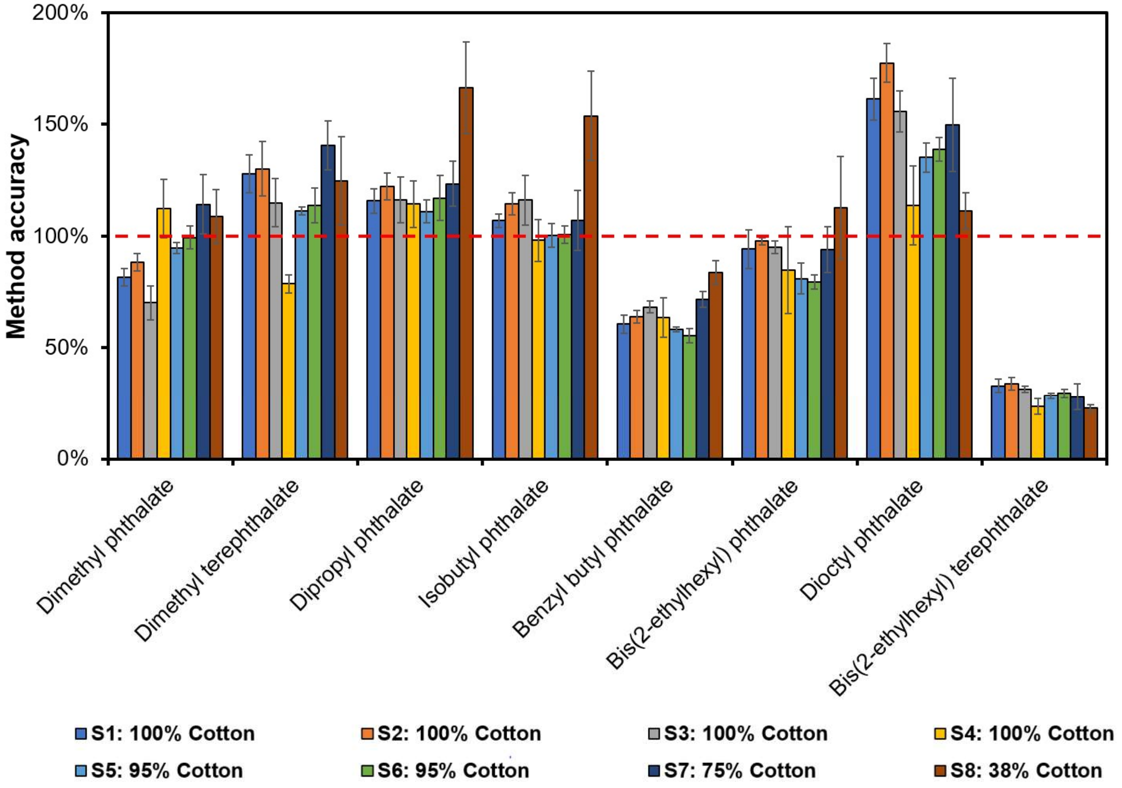

Figure S25: Additional compounds: Method accuracy “IS Response Method”

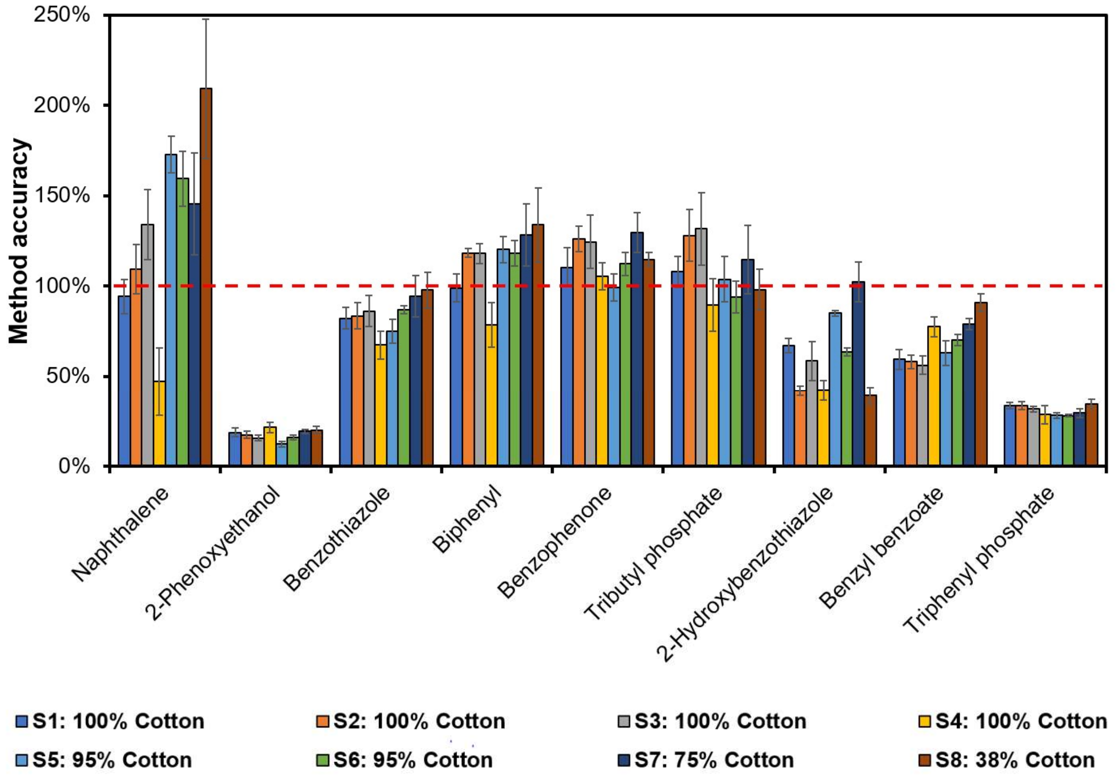

Figure S26: Reference standard spiked on textiles illustrating 60 target compounds

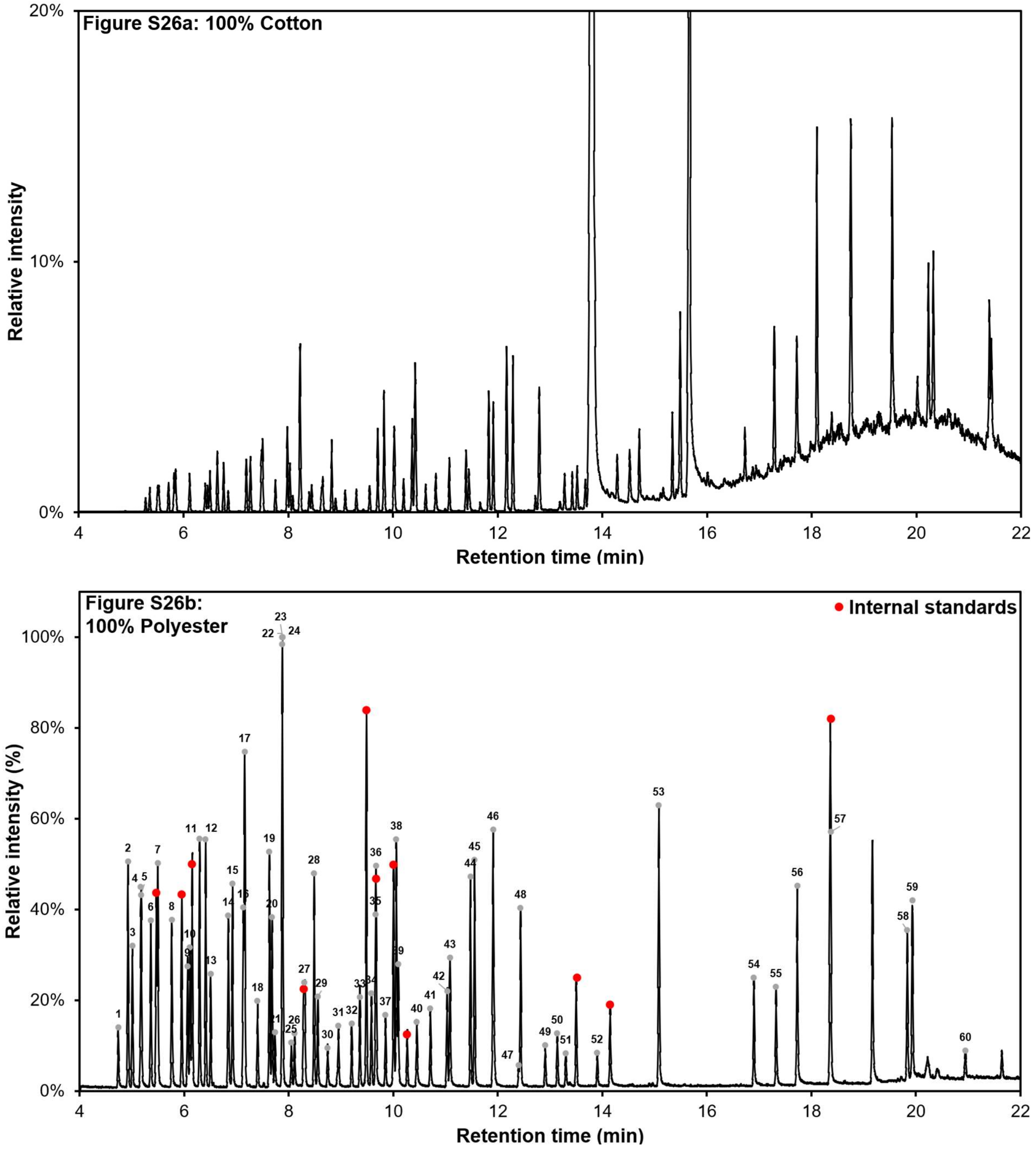

1. Catechol, 2. Naphthalene, 3. 4-Chloroaniline, 4. 2-Phenoxyethanol, 5. 2-Bromoaniline, 6. Benzothiazole, 7. Quinoline, 8. Isoquinoline, 9. 3-Bromoaniline, 10. 4-Bromoaniline, 11. 2-Methylquinoline, 12. 8-Methylquinoline, 13. 3,5-Dichloronitrobenzene, 14. 6-Methylquinoline, 15. 3-Methylquinoline, 16. 4-Methylquinoline, 17. Biphenyl, 18. 2-Nitroaniline, 19. 2,6-Dimethylquinoline, 20. 3,4-Dichloroaniline, 21. 1,4-Dinitrobenzene, 22. 2,4-Dimethylquinoline, 23. 1,3-Dinitrobenzene, 24. Dimethylphthalate, 25. 1,2-Dinitrobenzene, 26. 3-Nitrophenol, 27. 3-Nitroaniline, 28. Dimethylterephthalate, 29. 2,4-Dinitrophenol, 30. 4-Nitrophenol, 31. 2,5-Dinitrochlorobenzene, 32. 2,4-Dinitrochlorobenzene, 33. Diethylbenzyl phosphonate, 34. 2,6-Dichloro-1,4-benzenediamine, 35. 4-Chloro-2-nitroaniline, 36. 4-Nitroaniline, 37. 3,5-Dinitrobromobenzene, 38. Benzophenone, 39. Tributyl phosphate, 40. 2-Hydroxybenzothiazole, 41. 2-Chloro-4-nitroaniline, 42. 2,6-Dichloro-4-nitroaniline, 43. Dibenzylamine, 44. Dipropyl phthalate, 45. Benzylbenzoate, 46. Anthracene, 47. Tetraethyl ethylenediphosphonate, 48. Isobutyl phthalate, 49. 6-Chloro-2,4-dinitroaniline, 50. 2,6-Dibromo-4-nitroaniline, 51. 2,4-Dinitroaniline, 52. 2-Bromo-4,6-dinitroaniline, 53. Pyrene, 54. Benzylbutyl phthalate, 55.

Triphenyl phosphate, 56. Tris(2-ethylhexyl) phosphate, 57. Bis(2-ethylhexyl) phthalate, 58. Dioctyl phthalate, 59. Bis(2-ethylhexyl) terephthalate, 60. Benzo(*a*)pyrene
